# Comparative plastome analysis and taxonomic classification of snow lotus species (*Saussurea*, Asteraceae) in Central Asia and Southern Siberia

**DOI:** 10.1007/s10142-024-01309-y

**Published:** 2024-02-23

**Authors:** Nudkhuu Nyamgerel, Shukherdorj Baasanmunkh, Batlai Oyuntsetseg, Zagarjav Tsegmed, Gun-Aajav Bayarmaa, Georgy Lazkov, Elizaveta Pyak, Hee-Young Gil, Inkyu Park, Hyeok Jae Choi

**Affiliations:** 1 Department of Biology and Chemistry, Changwon National University, Changwon, Korea; 2 Department of Biology, School of Arts and Science, National University of Mongolia, Ulaanbaatar, Mongolia; 3 Institute for Biology, National Academy of Sciences, Bishkek, Kyrgyzstan; 4 Department of Botany, Tomsk State University, Tomsk, Russia; 5 Department of Forest Biodiversity and Herbarium, Korea National Arboretum, Pocheon, Korea

**Keywords:** *Saussurea*, *Amphilaena*, Snow lotus, Plastome, Phylogenomics

## Abstract

Four species of *Saussurea*, namely *S. involucrata*, *S. orgaadayi*, *S. bogedaensis*, and *S. dorogostaiskii*, are known as the “snow lotus,” which are used as traditional medicines in China (Xinjiang), Kyrgyzstan, Mongolia, and Russia (Southern Siberia). These species are threatened globally, because of illegal harvesting and climate change. Furthermore, the taxonomic classification and identification of these threatened species remain unclear owing to limited research. The misidentification of medicinal species can sometimes be harmful to health. Therefore, the phylogenetic and genomic features of these species need to be confirmed. In this study, we sequenced five complete chloroplast genomes and seven nuclear ITS regions of four snow lotus species and other *Saussurea* species. We further explored their genetic variety, selective pressure at the sequence level, and phylogenetic relationships using the chloroplast genome, nuclear partial DNA sequences, and morphological features. Plastome of the snow lotus species has a conserved structure and gene content similar to most *Saussurea* species. Two intergenic regions (*ndh*J–*ndh*K and *ndh*D-*psa*C) show significantly high diversity among chloroplast regions. Thus, ITS and these markers are suitable for identifying snow lotus species. In addition, we characterized 43 simple sequence repeats that may be useful in future population genetic studies. Analysis of the selection signatures identified three genes (*rpo*A, *ndh*B, and *ycf*2) that underwent positive selection. These genes may play important roles in the adaptation of the snow lotus species to alpine environments. *S. dorogostaiskii* is close to *S. baicalensis* and exhibits slightly different adaptation from others. The taxonomic position of the snow lotus species, confirmed by morphological and molecular evidence, is as follows: (i) *S. involucrata* has been excluded from the Mongolian flora due to misidentification as *S. orgaadayi* or *S. bogedaensis* for a long time; (ii) *S. dorogostaiskii* belongs to section *Pycnocephala* subgenus *Saussurea*, whereas other the snow lotus species belong to section *Amphilaena* subgenus *Amphilaena*; and (iii) *S. krasnoborovii* is synonymous of *S. dorogostaiskii*. This study clarified the speciation and lineage diversification of the snow lotus species in Central Asia and Southern Siberia.

**Supplementary Information:**

The online version contains supplementary material available at 10.1007/s10142-024-01309-y.

## Introduction

*Saussurea* DC. is one of the most diverse genera in the Asteraceae family, with approximately 460–490 herbaceous species that are mainly prevalent in the alpine habitats of the Himalayas and temperate regions of Asia (Chen 2015; Raab-Straube 2017; Xu et al. 2019). This genus merged in the early-middle Miocene within the Hengduan Mountains (South Asia) and dispersed northward due to the Himalayan uplift and adjacent mountain range formation (Chen 2015). As a result, 150 species are distributed in Central Asia, and 100 species are distributed in Eastern Asia (Lipschitz 1979; Wang et al. 2009; Baasanmunkh et al. 2022). The *Saussurea* genus is divided into four subgenera, *Saussurea* DC., *Eriocoryne* (DC.) Hook. f., *Amphilaena* (Stschegl.) Lipsch., and *Theodorea* (Cass.) Lipsch. and approximately 15 sections, based on morphological characteristics (Wang et al. 2009; Shi and Raab-Straube 2011; Zhang et al. 2021a). Among them, *Amphilaena* is particularly rich in medicinal resources, and comprises 39 taxa (Chik et al. 2015; Raab-Straube 2017; Chen et al. 2019). This subgenus has a glabrous stem, leafy bracts, and solitary capitula, which have many adaptive traits from multiple origins and arose through convergent evolution (Zhang et al. 2021a). However, this characteristic is a well-known strategy for adaptation to high altitudes and occurs in other angiosperms from several families (Omori et al. 2000).

Misidentified, rare, medicinally important species belonging to the subgenus *Amphilaena* are distributed in Central Asia and Southern Siberia. Particularly, *Saussurea bogedaensis* Yu J.Wang & J.Chen, *Saussurea involucrata* (Kar. & Kir.) Sch.Bip., *Saussurea orgaadayi* Khanm. & Krasnob., and *Saussurea dorogostaiskii* Palib. (or *Saussurea krasnoborovii* S.V.Smirn) poorly investigated based on morphological and molecular evidence. For instance, *S. orgaadayi* and *S. involucrata* have been distributed in the western and central parts of Mongolia for a long time; however, the taxonomic circumscription of these two species is difficult. Recently, another similar species, *S. bogedaensis*, was discovered, which was previously known as *S. involucrata* or *S. orgaadayi* (Chen and Wang 2018; Baasanmunkh et al. 2020). Moreover, the taxonomic statuses of *S. dorogostaiskii* and *S. krassnorobovii* remain unclear (Smirnov 2004; Raab-Straube 2017; Smirnov et al. 2018). In traditional medicine, the abovementioned species are known as “snow lotus or vansemberuu” and are used to treat lung diseases (Nyambayar et al. 2011; Chik et al. 2015; Norris 2020). However, given these species are used for medicinal purposes, misidentification can be fatal to humans (Chik et al. 2015). Thus, there is an urgent need to identify possible substitutes for these species.

Phylogenetic relationships of the subgenus *Amphilaena*, based on the nuclear ribosomal DNA (nrDNA) (ITS) and plastid (*mat*K, *rbc*L, *trn*K, *trn*H-*psb*A) barcoding markers, explored by Chen et al. (2019) showed that ITS and *rbcL* markers were well identified in all species. However, based on the chloroplast whole-genome (plastome) analysis of *Saussurea*, these chloroplast markers failed to resolve relationships across the genus (Zhang et al. 2019). Recently, some classifications and diversity patterns of *Saussurea* have been revised based on morphological and ecological traits and plastome (Xu et al. 2019; Zhang et al. 2019, 2021a, c). Chloroplasts are organelles that play an essential role in photosynthesis in green plants (Bruneau et al. 2007). They have a maternally inherited haploid genome and have the potential to significantly advance the resolution of evolutionary relationships among complex plant lineages (Ravi et al. 2008). They can be used to infer well-resolved phylogenetic relationships, even at the species level, and for species classification and population genetic studies (Jansen et al. 2007). Positive selection of chloroplast genes can also improve our knowledge of plants adapting to extreme environments in alpine regions (Bock et al. 2014). The application of divergent regions and simple sequence repeats (SSRs) as DNA barcodes can guarantee species identification (Shen et al. 2020). In addition to plastome data, nrDNA, which constitutes the catalytic core of ribosomes, is also widely used for phylogenetic studies in terrestrial plants (Rodnina et al. 2007). The 45S nrDNA units are composed of three subunits (18S, 5.8S, and 26S rDNAs) and two internal transcribed spacer regions (ITS1 and ITS2), while thousands of 45S units are tandemly repeated in the nuclear genome (Long and Dawid 1980). Owing to its structural advantages, this sequence is highly conserved, making it a useful resource for phylogenetic studies of terrestrial plants (Lagesen et al. 2007). Therefore, the plastome and nrDNA sequences and morphology features are necessary for identifying the snow lotus species.

This study aimed to characterize the plastome of snow lotus species in Central Asia and Southern Siberia, and identify genetically variable regions for the development of informative chloroplast DNA markers through comparisons with closely related species within the *Amphilaena* subgenus, to reconstruct the phylogenetic relationships among the snow lotus species. This study seeks to provide insight into the evolution of *Saussurea* species in Central Asia and Southern Siberia.

## Materials and methods

### Plant materials, DNA extraction, PCR amplification, and sequencing

In this study, fresh leaves from *S. bogedaensis*, *S. orgaadayi*, *S. dorogostaiskii*, and *S. involucrata* from the snow lotus group and *Saussurea baicalensis* (Adams) B.L.Rob. from Mongolia, Kyrgyzstan, and Russia, collected since 2016, were used (Table S1). We did not collect herbarium specimens of the snow lotus group from the wild because they are a globally threatened species. Moreover, we examined specimens from the herbarium of Komarov Botanical Institute RAS, Moscow University, Martin-Luther-Universität Halle-Wittenberg, Central Siberian Botanical Garden, Tomsk State University, and National Univesity of Mongolia, according to Thiers (2023). Detailed photographs of all species were taken by the authors during the field surveys. The distribution maps were created using ArcGIS (ESRI 2011) based on field collections and herbarium specimens.

Total genomic DNA was extracted from silica gel-dried leaf material using the CTAB method (Doyle and Doyle 1987). The nuclear ITS region (White et al. 1990) was used for the PCR amplification and Sanger sequencing. PCR amplification was performed using 100 ng/μl of the sample, as described in previously (Baasanmunkh et al. 2020). PCR products were sequenced in both directions by Macrogen (Seoul, Korea). Sequences were aligned using ClustalW (Thompson et al. 2003); BioEdit was used to set the default settings and perform manual adjustments (Hall et al. 2011). Ambiguous nucleotide bases were corrected using corresponding bases of sequences obtained using a reverse primer. We sequenced the plastome of one accession from each snow lotus species. The plastome library was prepared from the total genomic DNA using the TruSeq DNA Nano Kit, along with NextSeq 500 platform (Illumina, San Diego, CA, USA), following the manufacturer’s protocol. Trimmomatic v. 0.36 (Bolger et al. 2014) was used to remove adapter sequences and low-quality reads to reduce bias. The total number of bases and reads, GC content (%), and the Q20 (%) and Q30 (%) scores were calculated after filtering. A base quality plot generated using FastQC v. 0.11.5 (Antil et al. 2023) was used to check the overall quality of the data and shows the range of quality values for each cycle.

### Plastome assembly/annotation

NOVOplasty v.4.1.0 was used to perform de novo assembly using various k-mers (Nicolas et al. 2017). The best k-mer was selected based on the assembly results, including the number of contigs, total length of contigs, and N50. The raw data reads were mapped to an assembly result to identify the insert size of the raw data and number of reads used in the assembly. The assembly and orientations were confirmed using BLAST tool of NCBI (https://www.ncbi.nlm.nih.gov/) and graphic views using Geneious Prime® 2023.2.1 (https://www.geneious.com; Biomatters Ltd.) and Geseq—annotation of organellar genomes (Tillich et al. 2017). The transfer RNAs (tRNAs) were identified using tRNA-scan-SE (Lowe and Chan 2016). A circular chloroplast genomic map was visualized using Chloroplot online software (https://irscope.shinyapps.io/Chloroplot/) (Zheng et al. 2020).

### Plastome analysis

The pairwise whole plastome alignment was visualized using MAUVE v2.3.1 (Darling et al. 2010). The boundary shifts in the single copy (SC)/inverted repeat (IR) at four junctions of the plastomes were compared using IRscope (https://irscope.shinyapps.io/irapp/) (Amiryousefi et al. 2018). The mVISTA program (Frazer et al. 2004) in the Shuffle-LAGAN mode (Brudno et al. 2003) was used to detect species-specific genetic variation by comparing newly sequenced plastomes, with *Saussurea obvallata* (DC.) Sch.Bip. as a reference.

### Repeat sequence analysis

We used REPuter to identify forward, reverse, palindromic, and complementary repeats with a minimum length of 20 bp, 90% identity, and a Hamming distance of 3 (Kurtz 2001). SSRs were detected using MISA (Beier et al. 2017), with the minimum number of repeat parameters set to 10, 5, 4, 3, 3, and 3 for mononucleotides, dinucleotides, trinucleotides, tetranucleotides, pentanucleotides, and hexanucleotides, respectively. Tandem repeats ≥ 20 bp were identified using the tandem repeat finder (Benson 1999) with a minimum alignment score of 50 and a maximum period size of 500; the identity of repeats was set to ≥ 90%.

### Genome divergence

The five newly sequenced samples, *S. involucrata*, *S. orgaadayi*, *S. bogedaensis*, *S. dorogostaiskii*, and *S. baicalensis*, and four species from the NCBI database were used in this experiment. The plastomes was individually aligned using MAFFT ver. 7.388 (Katoh 2002). Sliding window analysis was used to calculate the nucleotide variability (Pi) among the plastomes using DnaSP v.6.11 (Rozas et al. 2017). The step size was set to be 200 bp, with a 600-bp window length. Protein-coding genes (coding regions), intergenic spacers, and introns (non-coding regions) of the plastomes were extracted separately to screen for polymorphic hotspots. The relative synonymous codon usage (RSCU) of the plastomes was analyzed using the MEGA11 software (Tamura et al. 2021). The codon usage distribution of snow lotus plastomes was visualized using the Heatmapper tool with average linkage clustering and Euclidean distance measurement methods (Babicki et al. 2016). An RSCU < 1.00 indicated a codon that was used less frequently than expected, whereas an RSCU > 1.00 indicated a codon that was used more frequently than expected. Selective pressure was analyzed for consensus exons of the protein-coding genes in the snow lotus species using KaKs_Calculator 2.0 (Wang et al. 2010) with calculation mode NY and genetic code 11 (bacterial and plant plastid code), using *S. obvallata* as a reference. Genes with a Ka/Ks > 1 ratio were considered to be under positive selection, and genes with a Ka/Ks ratio < 1 were considered to be under purifying selection.

### Phylogenetic analysis

The nrDNA ITS region and plastome sequences of the *Saussurea* species included in this study were used to determine the phylogenetic position of the snow lotus species within the *Saussurea* genus. Plastome alignment datasets were filtered to remove ambiguously aligned regions using GBlocks ver. 0.91.1 (Talavera and Castresana 2007). The best-fitting model for nucleotide substitutions was determined using the Akaike information criterion in jModelTest v2.1.10 (Darriba et al. 2012), and the GTR + I + G model was selected for maximum likelihood (ML) analysis. The GTR + G model was selected for the Bayesion inference (BI) analysis. The maximum parsimony (MP) analysis was conducted using PAUP* v4.0b10 (Swofford and Documentation 1989); the searches included 1000 random addition replicates and tree bisection and re-connection branch swapping using the MulTrees option. ML analysis was performed using RaxML v. 8.0.5 (Stamatakis 2014), with 1000 bootstrap replicates. BI analysis was performed using MrBayes 3.2.2 (Ronquist et al. 2012), with two independent runs of four simultaneous chains, executed for 5,000,000 generations using the Markov chain Monte Carlo algorithm. Trees were sampled every 5000 generations, and the first 25% were discarded as burn-in. The trees were determined using a 50% majority-rule consensus to estimate posterior probabilities (PPs). The reconstructed trees were visualized using FigTree v.1.4.2 (Rambaut 2012).

## Results

### Comparison of the morphological characteristics of the snow lotus species

**Fig. 1:**
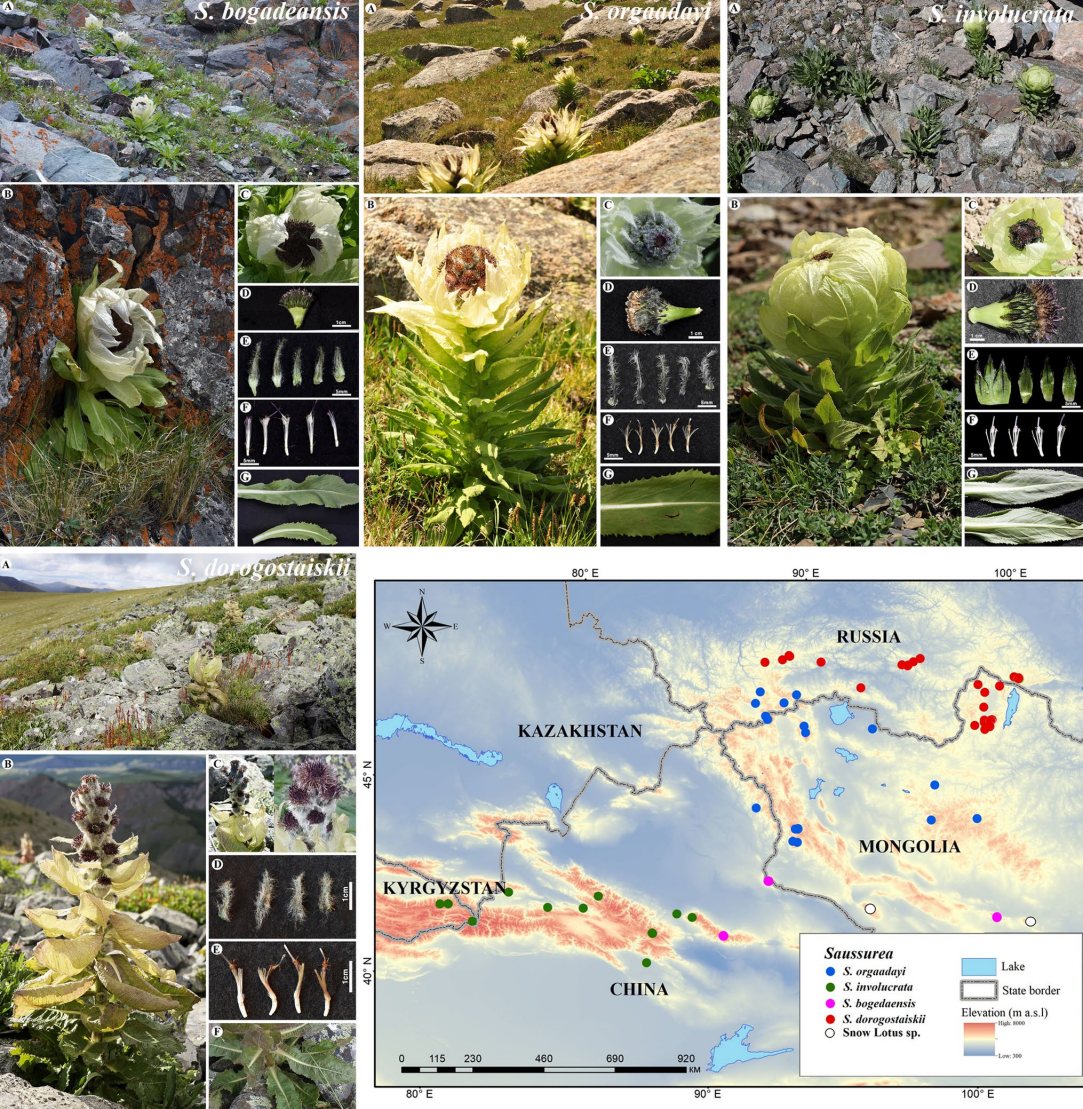
Morphological characteristics and geographic distribution map of snow lotus species in Central Asia and Southern Siberia. **A** General habitat. **B** Adult flower. **C** Inflorescences. **D** Disk flower. **E** Phyllaries. **F** Pappus. **G** Leaves. (Photo credit: S. Baasanmunkh and H.J. Choi)

Morphological differences among the snow lotus species are presented in Table 1 based on field observations and previous taxonomic studies, along with their general distribution and conservation status. In addition, we provided taxonomic keys and photo illustrations of snow lotus species, including habitats and closeups of the florets, pappus, phyllaries, and leaves (Fig. 1). Snow lotus species can be clearly distinguished based on their morphological characteristics. Moreover, we addressed the taxonomic notes of *S. dorogostaiskii* and *S. krassnoborovii* which are previously unresolved species. Based on the field surveys conducted in this study, in the early stages of flowering, the synflorescence was surrounded by pale green leaves, resembling cabbage. The synflorescence rachis became elongated and racemiform in mid-July after opening, and the uppermost leaves turned yellow, membranous, and boat-shaped. After late July, all the uppermost leaves withered. After pressing the specimens, membranous bracts were removed.

**Table 1:** Comparison of the morphological characteristics of the snow lotus species in Central Asia and Southern Siberia

Taxon	*S. dorogostaiskii*	*S. orgaadayi*	*S. involucrata*	*S. bogedaensis*
Inflorescense	Racemiform	Corymbiform	Corymbiform	Corymbiform
Capitula no	10–20	20–30	10–20	15–30
Bract	Ovate-narrowly ovate, boat- shaped, strongly keeled, cream-yellow	Triangular-ovate, apex long acuminate	Ovate-elliptic, apex acute	Ovate-elliptic, apex acute
Phyllary	Narrowly triangular or triangular-acuminate, apex acute, phyllaries densely pubescent throughout	Linear-subulate, apex long acuminate, phyllaries densely pubescent throughout	Triangular-ovate, apex acute or obtuse, phyllaries glabrous, rarely sparsely pubescent apically or along midvein	Subulate to acuminate, phyllaries densely pubescent middle-upper part
Involucre	Broadly campanulate	Campanulate	Hemispheric	Campanulate
Pappus color	Dirty white-yellowish	Straw-colored	Dirty white	Dirty white
Stem leaves	Elliptic to ovate, tinged with purple to yellowish, apex obtuse	Lanceolate, apex long acuminate	Narrowly ovate, elliptic, or obovate, apex acute,	Elliptic, apex obtuse
Basal leaves	Yellowish brown	Yellowish brown, stripes up to 1 cm wide	Dark brown, stripes up to 2–3 mm wide	Dark brown, stripes up to 2–3 mm wide
Stem	25–100 cm	40–65 cm	10–15 cm	15–50 cm
Distribution	Mongolia (Khuvsgul) and Russia (Tuva)	China (Xinjiang Altai), Mongolia (Mongolian Altai and Khangai), and Russia (Altai)	China and Kyrgyzstan (Western Tianshan)	China (Eastern Tianshan), Mongolia (Dzungarian Gobi and Gobi-Altai)
Conservation status	Critically endangered	Endangered	Endangered	Critically endangered

### Key to the snow lotus species

- 1.Capitula 5–30, strongly condensed corymbiform, sessile or with up to 3-cm-long peduncles; uppermost stem leaves stellate spreading
- +Capitula 10–20, in racemiform, elongated synflorescence
- 2.Stem leaves lanceolate, apex long acuminate, with very dense glandular hair. Petiole remains of basal leaves with yellowish-brown strips up to 1 cm wide. Pappus straw-colored; phyllary triangular-ovate, apex acute or obtuse
- ***S. orgaadayi***
- +Stem leaves narrowly ovate, elliptical, or obovate. Petiole remains of basal leaves with dark brown strips up to 2–3 mm wide. Bracts ovate elliptic, apex acute. Pappus dirty white colored
- 3.Stem leaves apex acute. Phyllaries triangular-ovate, glabrous, rarely sparsely pubescent apically or along the midvein
- ***S. involucrata***
- +Stem leaves elliptical, apex obtuse. Phyllaries subulate to acuminate, densely pubescent middle-upper part
- ***S. bogedaensis***
- 4.Leaves oblong-elliptical to obovate, densely covered with glandular hair; upper stem leaves membranous, pale yellow, boat-shaped with strongly keeled, stellate surrounding synflorescence before flowering
- ***S. dorogostaiskii***
- +Leaf blade narrowly ovate to elliptical, apex acute to acuminate, both surfaces light green, abaxially glabrous, adaxially sparsely villous to subglabrous. Synflorescence not enclosed by the uppermost leaves. Pappus straw colored
- ***S. baicalensis***

### Assembly of plastome and ITS sequences

**Fig. 2:**
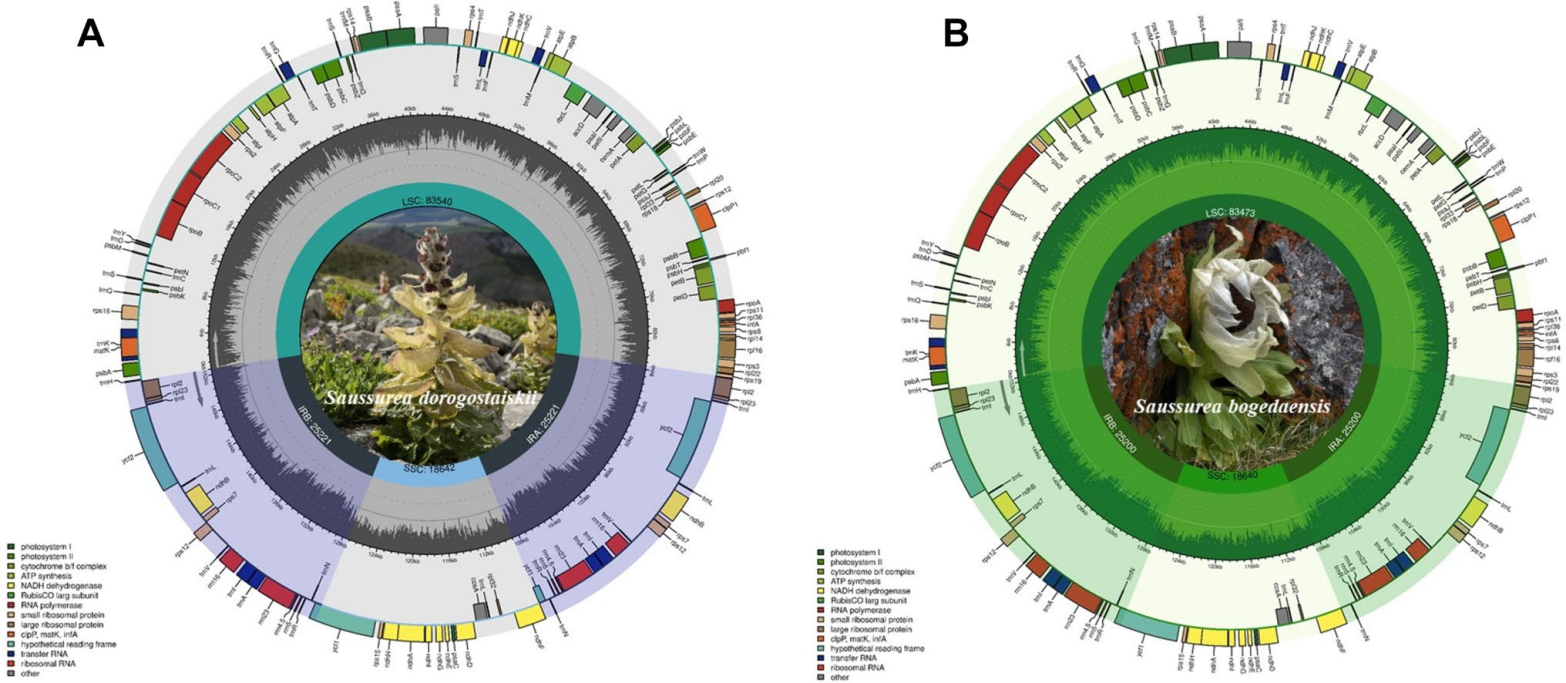
Circular map of the complete chloroplast genome of **A** *Saussurea dorogostaiskii* and **B** *Saussurea bogedaensis*. The inner ring is divided into four areas, clockwise, and includes the SSC, IRb, LSC, and IRa. The genes in the outer ring region are transcribed clockwise, while those in the inner ring are transcribed counterclockwise. In addition, this figure also reflects the GC content; the inner ring in dark gray or green indicates the GC content, and the light gray or green indicates the AT content. In the lower left is a legend that classifies cp genes according to their functions

The plastomes of *S. bogedaensis* and *S. dorogostaiskii* were sequenced from Mongolia for the first time, and *S. involucrata*, *S. orgaadayi*, and *S. baicalensis* were resequenced from a new origin in Kyrgyzstan and Mongolia. De novo assembly generated a single contig for each sample (Table S2). The snow lotus plastomes exhibited a double-stranded circular DNA molecule 152,512–152,624 bp long (Table 2). It had a natural quadripartite structure that comprised a large single copy (LSC), a pair of inverted repeats (IRs), and a small single copy (SSC) (Fig. 2). The GC content in the plastomes was 37.7%, whereas the GC contents in the IR, LSC, and SSC regions were 43.1, 35.8, and 31.4–31.3%, respectively (Table 2). The snow lotus plastomes encoded 109 unique genes, including 4 rRNA, 25 tRNA, and 80 protein-coding (CDS) genes; among these, 4 rRNA genes, 7 tRNA genes, and 7 CDSs were duplicated in the IR regions (Table S3). They harbored 18 intron-containing genes, among which 16 had a single intron and 2 had two introns (Table S4). Four intron-containing genes were found to be duplicated in the IR region. The *rps*12 gene was a trans-spliced gene with the 5′ end located in the LSC and the 3′ end located in the IR.

**Table 2:** Summary of the characteristics of the *Saussurea* plastomes

Species		*S. bogedaensis*	*S. dorogostaiskii*	*S. involucrata*	*S. orgaadayi*	*S. baicalensis*
Size (bp)/GC content (%)	Total	152,513/37.7	152,624/37.7	152,512/37.7	152,594/37.7	152,624/37.7
	LSC	83,474/35.8	83,541/35.8	83,526/35.8	83,562/35.8	83,541/35.8
	IR	25,199/43.1	25,221/43.1	25,193/43.1	25,194/43.1	25,221/43.1
	SSC	18,640/31.3	18,642/31.4	18,599/31.3	18,645/31.3	18,642/31.4
Number of genes	Total	131	131	131	131	131
	CDS	80	80	80	80	80
	tRNA	30	30	30	30	30
	rRNA	4	4	4	4	4
Accession number		OR426627	OR426626	OR426625	OR426629	OR426628

We newly sequenced 7 ITS sequences of snow lotus species, ranging from 650 to 700 bp in length. The final annotated plastome and ITS sequences were deposited in GenBank (Table 2 and S1).

### Comparative analysis of the snow lotus plastome

In snow lotus plastomes, the genome structure, gene order, and boundaries between IRs and single-copy regions were similar. Co-linearity in gene placement among the five newly sequenced snow lotus plastomes was assessed using MAUVE (Fig. S1). Gene order and locations were conserved; however, the LSC region had a 20-kb inversion (between *trn*S-GCU and *trn*G-UCC). Additionally, another 3-kb translocation (between *trn*S-GCU and *trn*E-UUC) was detected within the snow lotus plastome. The sequence identities of the snow lotus were analyzed using mVISTA, with the *S. obvallata* (MH926128) plastome serving as a reference (Fig. S2). As expected, genic regions were more conserved than intergenic regions. This pair of IR regions was highly conserved, followed by the LSC and SSC regions. In snow lotus, IR lengths ranged from 25,193 to 25,220 bp, and the borders between the IR regions and the two single copy regions (LSC and SSC) were similar. The *rps*19 and *ycf*1 genes spanned the LSC/IRb and SSC/IRa junctions, respectively (Fig. S3).

The RSCU was computed for the 80 protein-coding genes of the snow lotus species. These CDSs contained 22,793–22,814 codons and encoded 20 amino acids in the plastome (Fig. S4A). Among them, leucine (10.53%) was the most frequently detected amino acid, whereas cysteine (1.09%) was the least ubiquitous (Fig. S4B). To identify codon patterns, we analyzed the codon distribution in eight plastomes. Snow lotus species exhibited similar patterns in 23 codons, and other codons have two types of patterns, with high RSCU values for AGA (arginine), TTA (leucine), and GCT (alanine) (Fig. S5). The first pattern is represented in *S. bogedaensis*, *S. involucrate*, and *S. orgaadayi*; other pattern is represented in *S. dorogostaiskii* and *S. baicalensis*. Almost all CDSs in snow lotus species have the standard ATG start codon, but *psb*L started with TGC/ACG. Codons with A or T in the third position had a strong codon bias. Among the three stop codons, TGA was the most commonly detected.

### Distribution of repeat sequences

**Fig. 3:**
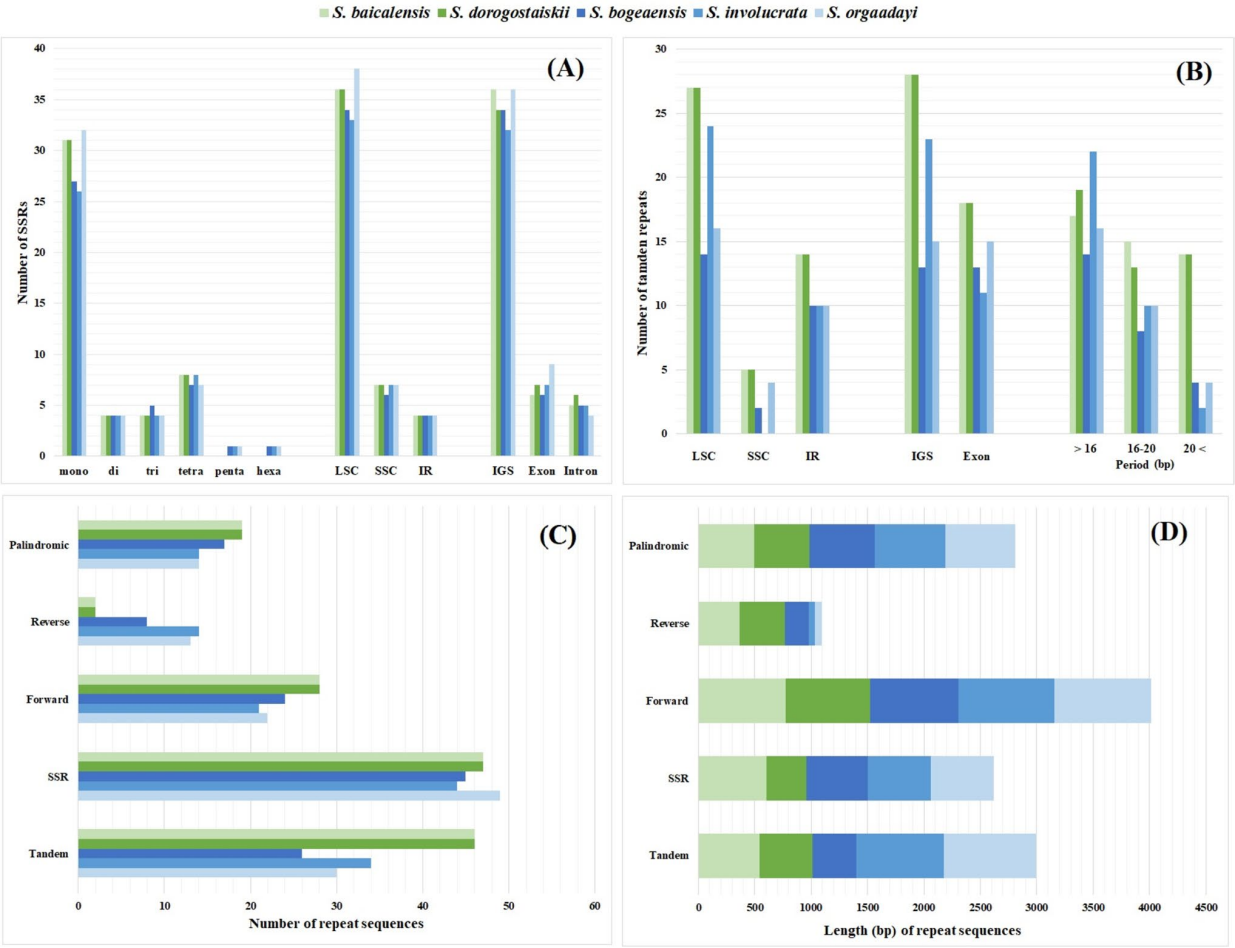
The distribution of repeat sequences in of snow lotus species and *S. baicalensis* plastomes. **A** The number of simple sequence repeat (SSR) motifs and the distribution of SSRs in the genome regions. **B** The number of repeat sequences, distribution of tandem repeats in the genome regions, and length of motifs. **C** Total number of repeat sequences of plastome. **D** Total length (bp) of repeat sequences of plastome

We investigated the repeat sequences of five *Saussurea* species to determine the characteristics and proportions of repeat sequences within the snow lotus group. The snow lotus species had a variable number of repeats, including forward, reverse, and palindromic repeats. For SSRs, mononucleotide motifs were the most abundant in all species, followed by tetranucleotide motif repeats (Fig. 3A). A total of 44–49 SSRs were identified, mostly in the LSC region, particularly within the intergenic spacer (IGS) region. Mononucleotide SSRs (25–31) mostly contained A (9–14), T (16–19), and C (1) repeat units. We found 26–46 tandem repeats that were generally 9–27 bp long (Fig. 3B). Among the protein-coding genes, *rbc*L and *ycf*2 commonly contain tandem repeats in the snow lotus plastomes. The 5′ end of the *ndh*B gene of *S. baicalensis* and *S. dorogostaiskii* included a 24-bp repeated insertion. A higher number of SSRs and tandem repeat sequences were observed in *S. orgaadayi* and *S. dorogostaiskii*, respectively (Fig. 3C). The snow lotus plastome included a higher number of forward repeats, whereas it had the lowest number of reverse repeats and no complementary repeats (Fig. 3D).

### Estimation of genetic variability

**Fig. 4:**
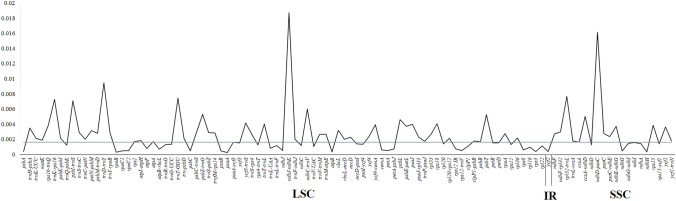
Comparison of the nucleotide diversity (Pi) values among the snow lotus species. The mean Pi value indicated by blue line

We analyzed the genetic divergence of genes and intergenic regions within the snow lotus plastomes. Overall, the snow lotus plastomes had an average genetic diversity (Pi) value of 0.0016. The highest Pi values were observed in IGS regions *ndh*J–*ndh*K (0.018) and *ndh*D*-psa*C (0.016) in the LSC and SSC regions, respectively. Genic and intergenic regions in the IR regions were relatively constant (Fig. 4).

### Molecular evolution of snow lotus

**Fig. 5:**
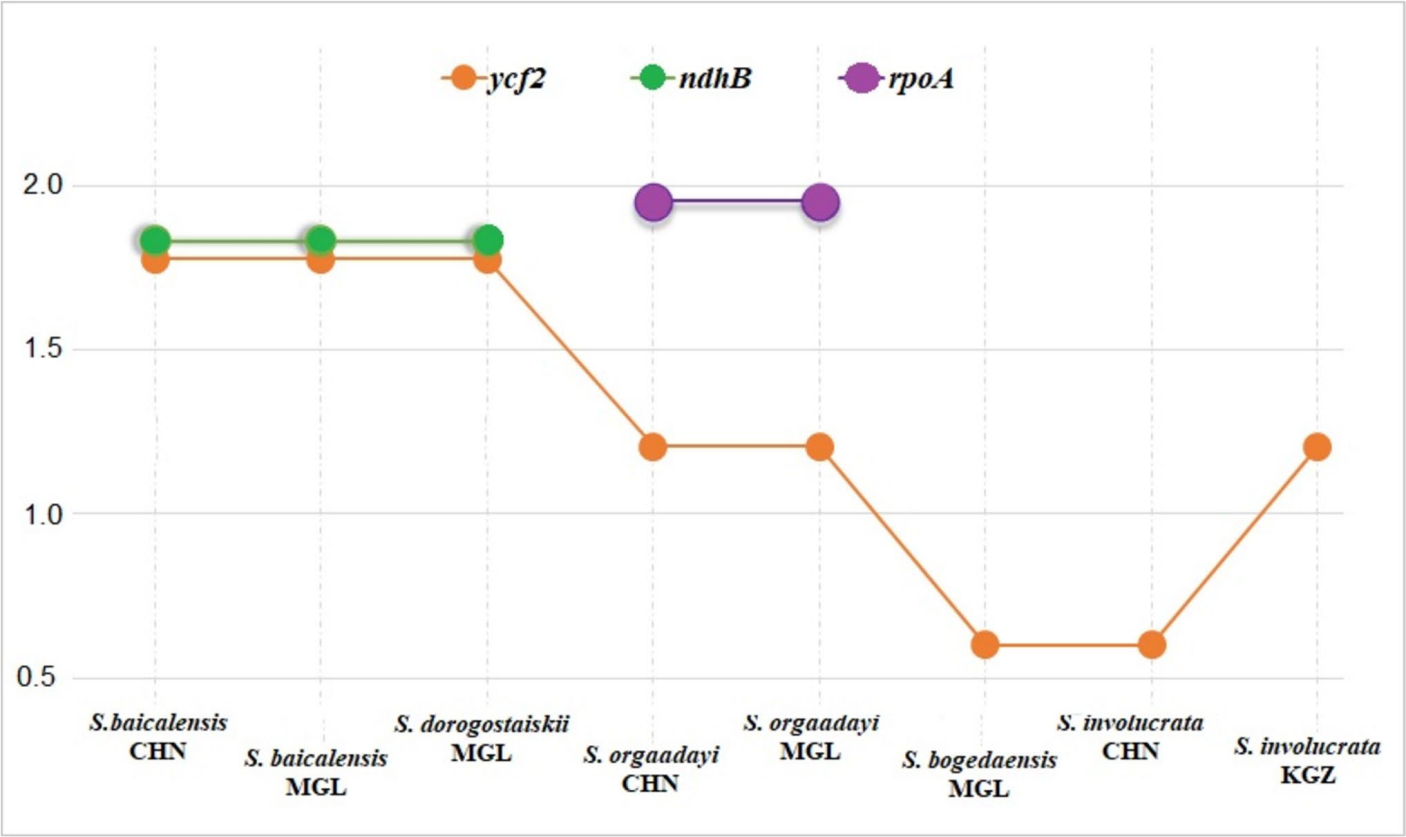
Positively selected genes (Ka/Ks > 1) in the snow lotus species and *S. baicalensis*

A total of 80 consensus protein-coding genes from eight snow lotus samples were evaluated to assess selective pressure, and compared with *S. obvallata*. Three genes were found to have undergone positive selection (Fig. 5): *ycf*2 had undergone positive selection in most snow lotus species, except *S. bogedaensis* MGL *and S. involucrata* MGL; *ndhB* (1.83) was found in *S. baicalensis* MGL and *S. dorogostaiskii* MGL; *rpo*A (1.95) was found in *S. orgaadayi*. Fifteen genes were found to be under purifying selection in snow lotus species. Some of them show specific selection in a single species: *psb*T in *S. involucrata* KGZ, *rps*2 and *ycf*4 in *S. baicalensis* CHN, and *ccs*A in *S. bogedaensis* MGL. Furthermore, synonymous substitution (Ks) was represented in 21 genes, with the highest values in *ndh*B and *rpo*C2 in the *S. baicalensis* and *S. dorogostaiskii*, and *ndh*F and *psb*B in *S. involucrata* from Kyrgyzstan (Table S5). The *psa*A, *psb*C, *ndh*B, *ndh*I, and *rpo*C2 had specific mutations in S. *dorogostaiskii* and *S. baicalensis* plastomes. Moreover, *psa*B, *psb*B, and *ndh*D included specific mutations in the *S. bogeadensis*, *S. involucrata*, and *S. orgaadayi* plastomes. In the 43 genes that included non-synonymous substitutions (Ka), high values were noted in *ndh*B and *rpo*C2 of *S. baicalensis* and *S. dorogostaiskii*, and *psb*B of *S. involucrata* from Kyrgyzstan (Table S6). Among the noticed genes, six (*ndh*J, *psa*A, *psb*A, *psb*H, *rps*4, and *ycf*2) exhibited the same substitution in all species; eight (*acc*D, *atp*B, *mat*K, *psa*C, *rbc*L, *rpo*C1, *rpo*C2, and *ycf*1) were highly mutated in *S. baicalensis* and *S. dorogostaiskii*; nine (*ndh*A, *ndh*B, *psa*J, *rps*15, *rps*16, *rpl*20, *rpl*33, and *ycf*4) included mutation in only *S. baicalensis* and *S. dorogostaiskii*; six genes (*psa*B, *psb*B, *psb*L, *psb*T, *ndh*D, *rpl*22) include mutations in only *S. bogeadensis*, *S. involucrata* and *S. orgaadayi*; and other genes included species-specific mutations.

### Phylogenic position of snow lotus group within *Saussurea*

**Fig. 6:**
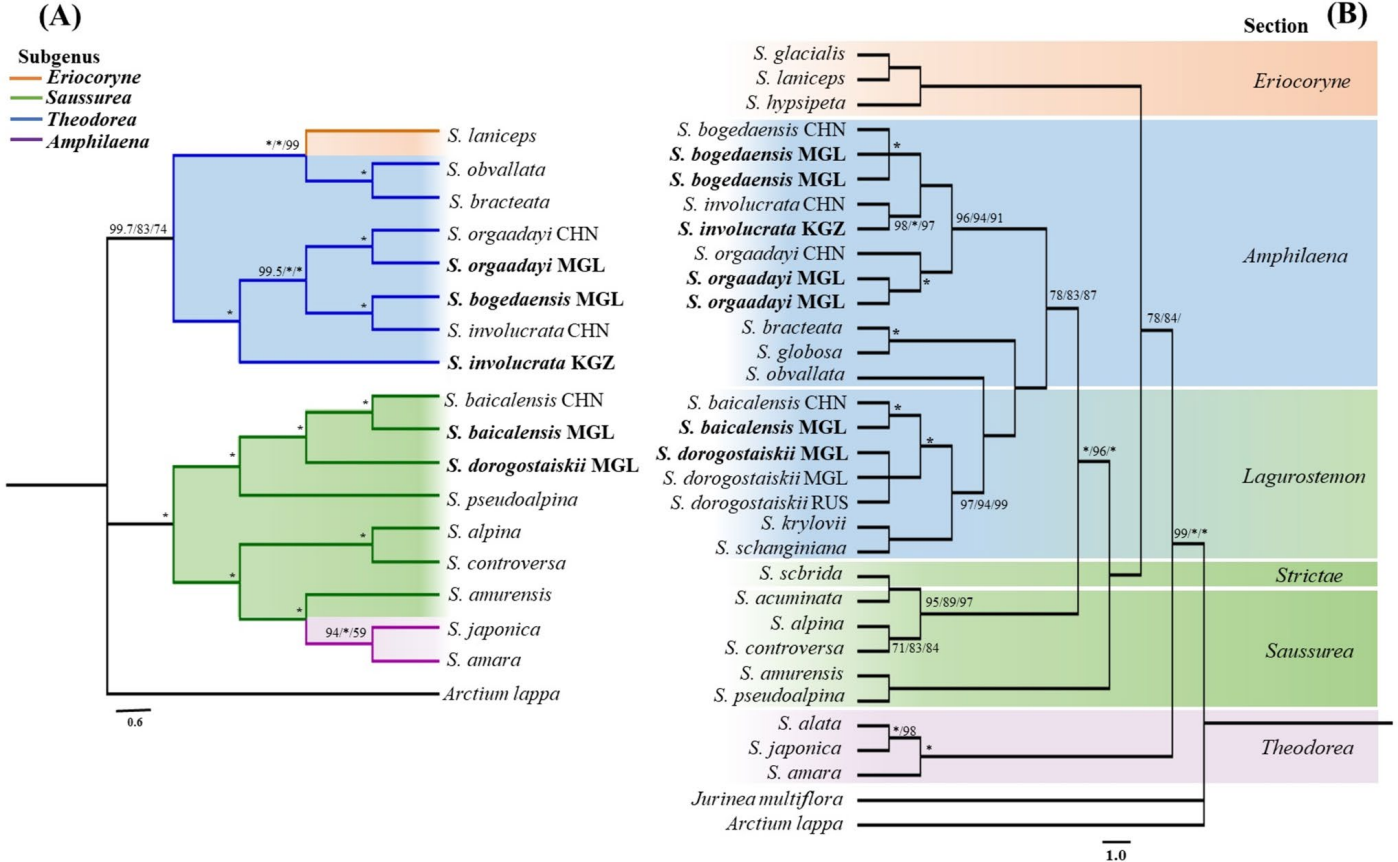
Phylogenetic tree based on **A** plastome and **B** nrDNA. Bayesian inference (BI), maximum likelihood (ML) and maximum parsimony (MP) support values indicated at each branch, following the order: BI/ML/MP. Maximum support values are labeled by a star. Black colored numbers indicate nodes constrained with fossils in the analysis. The newly sequenced samples are in bold. The samples from China, Kyrgyzstan, and Mongolia are abbreviated as CHN, KZS, and MNG, respectively. The branch of subg. *Ericoryne*, subg. *Saussurea*, subg. *Amphilaena*, and subg. *Theodorea* are represented by orange, green, blue, and purple color, respectively

Two datasets (plastome and nrDNA) were compiled to verify the phylogenetic position of the snow lotus species. The datasets contained 19 plastomes and 32 ITS sequences of *Saussurea* species from four different subgenus according to Raab-Straube (2003), Kita et al. (2004), Wang et al. (2007; 2010), Chen and Wang (2018), Chen et al. (2019), and Baasanmunkh et al. (2020), including those sequenced samples in this study (Table S7). *Jurinea multiflora* (L.) B.Fedtsch. and *Arctium lappa* L. were used as outgroups. The plastome alignment was 156,494 bp long, of which 154,497 bp were constant and 633 bp were parsimony-informative. The ITS alignment contained 442 bp, including 283 and 76 bp with constant and parsimony-informative characteristics, respectively. The newly sequenced *S. dorogostaiskii* samples from both Mongolia and Russia (previously named *S. krasnoborovii*) formed a highly supported monophyletic clade (BP = 100%); *S. orgaadayi* samples from Khangai (previously named *S. involucrata*) and Mongolian Altai in Mongolia were identified as the same species as *S. orgaadayi* from Xinjiang, China; and *S. bogedaensis* samples from Gobi-Altai (previously named *S. involucrata*) and Dzungarian Gobi regions in Mongolia were identified as the same species as *S. bogedaensis* from Xinjiang, China (Fig. 6). The topologies of the BI, ML, and MP analyses were highly congruent with the same dataset and supported by strong bootstrap values and PPs. The phylogenetic positions of the sections within *Saussurea* were not consistent among the phylogenetic tree of plastomes and nrDNAs. *S. bogedaensis*, *S. involucrata*, and *S. orgaadayi* were clustered with section *Amphilaena* species; *S. dorogostaiskii* and *S. baicalensis* clustered together under subgenus *Saussurea* based on the plastome sequences (Fig. 6A). Whereas in the ITS phylogenetic tree, the snow lotus species exhibited a monophyletic pattern (Fig. 6B).

## Discussion

### Classification and distribution of the snow lotus species

Taxonomic identification of *Saussurea* is notoriously difficult because of the high diversity of specialized morphology traits that have developed to adapt them to the different environmental stresses experienced in mountainous regions (Wang et al. 2009; Zhang et al. 2019; Zhang et al. 2021a, c). For example, *Amphilaena* is a non-monophyletic group based on DNA barcoding and plastome data (Wang et al. 2009; Xu et al. 2019). Recent whole-genome sequencing techniques and the sampling of threatened species have increased the availability of DNA barcoding (Li et al. 2019). In this study, we investigated the classification of snow lotus species by comparing our findings with those of previous studies. In particular, some specimens that were previously identified as *S. dorogostaiskii* (Smirnov 2004) were identified as *S. krasnoborovii* in this study; hence, we preserved the name *S. dorogostaiskii* for populations from Mongolia without membranous, pale-yellow upper stem leaves. More recently, *S. krasnoborovii* has been classified as the same as *S. dorogostaiskii* based on morphological characteristics (Raab-Straube 2017). Later, Smirnov et al. (2018) updated the checklist of *Saussurea* in Eurasia, which classified *S. dorogostaiskii* and *S. krasnoborovii* as different species. During our field surveys, we found various habitat types of *S. dorogostaiskii* in Mongolia and Russia at different periods, all of which were identified as single species based on their morphology, plastome, and nrDNA sequences (Table 1 and Figs. 1 and 6). Our observations confirmed the results of Raab-Straube (2017) that *S. krasnoborovii* is synonym of *S. dorogostaiskii*. This species is distributed in the Buryat and Tyva of Russia, and the Khuvsgul region of Mongolia (Fig. 1K). This species grows on both open and forested mountains between 1500 and 2500 m, primarily in and around boulders or talus patches, and sometimes within smaller scree rocks (Norris 2020).

*Saussurea baicalensis* is a type species of section *Pycnocephala* subgenus *Saussurea* (Shi and Raab-Straube 2011), which is closely related to *S. dorogostaiskii* (Fig. 6). Initially, *S. dorogostaiskii* shared a morphology similar to *S. baicalensis* (Palibin 1928) and was classified in the same section as *S. baicalensis* based on morphology (Smirnov 2004). Raab-Straube (2017) later discovered that *S. dorogostaiskii* occupied an intermediate position between the section *Amphilaena* subgenus *Amphilaena* and section *Pycnocephala* (*Lagurostemon*) subgenus *Saussurea* (Table 3). Consequently, *S. dorogostaiskii* belongs to section*. Pycnocephala* with *S. baicalensis* based on their genetic and morphological features. Further studies focusing on determining the position of section *Pycnocephala* within this genus are required, because the phylogenetic position of section *Pycnocephala* on the plastome and nrDNA trees is not consistent. The section *Pycnocephala* was clustered with subgenus *Saussurea* species on the plastome tree (Fig. 6A), and with subgenus *Amphilaena* species in the nrDNA tree (Fig. 6B). This section may have an intermediate position between the subgenuses *Amphilaena* and *Saussurea*.

**Table 3:** Classification of *S. dorogostaiskii* and related species within the sections and subgenus of *Saussurea*

*S. dorogostaiskii*	*S. baicalensis*	*S. krasnoborovii*	Reference
	sect. *Pycnocephala*		Lipschitz (1979)
sect. *Pycnoñephala*	sect. *Pycnoñephala*	sect. *Amphilaena*	Smirnov (2004)
sect. *Amphilaena*	sect. *Lagurostemon* = *Pycnoñephala*	syn of *S. dorogostaiskii*	Raab-straube (2017)
sect. *Lagurostemon*	sect. *Lagurostemon*	sect. *Amphilaena*	Smirnov et al. (2018)
**sect. *Pycnoñephala***	sect. *Pycnoñephala*	**syn of *S. dorogostaiskii***	This study

The morphological features of *S. orgaadayi*, *S. involucrata*, and *S. bogedaensis* are very similar, such as bracts surrounding the corymbiform synflorescence, cream-yellow bracts that aggregate below the florescence, and hollow stem ≥ 1.5 cm in diameter near the base (Fig. 1) (Shi and Raab-Straube 2011; Raab-Straube 2017; Chen and Wang 2018). However, they are significantly differentiated by the shape of the bract, pappus, and leaf margin (Table 1) (Chen and Wang 2018). Based on our molecular and morphological analysis, we confirmed the distribution of *S. bogedaensis* in the Gobi-Altai region and *S. orgaadayi* in the Khangai region of Mongolia, where *S. involucrata* was previously observed (Grubov 1982). The samples from the Khangai and Altai Mountains present straw-colored pappus, triangular-ovate bracts with a long acuminate apex, and phyllaries densely pubescent throughout and with up to 60-cm-tall stems, which are typical characteristics of *S. orgaadayi* rather than *S. involucrata* (Shi and Raab-Straube 2011). The samples from Gobi-Altai presented elliptic stem leaves with an obtuse apex and dirty white-colored pappus, which are typical characteristics of *S. bogedaensis* (Chen and Wang 2018). As a result, *S. involucrata* was excluded from the Mongolian flora because of long-term misidentification as *S. orgaadayi* or *S. bogedaensis*. In addition, recently published studies have suggested that *S. involucrata* were distributed in the western Tianshan Mountains in China and Kyrgyzstan, *S. bogedaensis* in eastern Tiansan in China, Dzungarian Gobi and Govi-Altai regions in Mongolia, and *S. orgaadayi* in the Altai Mountains in China, Mongolia, and Russia (Fig. 1K) (Raab-Straube 2017; Chen and Wang 2018; Chen et al. 2019; Baasanmunkh et al. 2020; Erst et al. 2022).

### Plastome feature and diversity

The complete plastome of *Saussurea* has recently been studied (Xu et al. 2019; Zhang et al. 2019, 2021a, c). However, previous studies have not explored snow lotus species in Central Asia and Southern Siberia, and there remains a gap for a better understanding of their relationships and evolutionary patterns. In this study, we sequenced and characterized the plastomes of snow lotus species in Central Asia and Southern Siberia. The complete plastome of the snow lotus is a typical quadripartite structure with one LSC, one SSC, and two IR regions (Fig. 2), which is a highly conserved pattern in green plants (Bruneau et al. 2007). Plastome genome size and gene order and number are conserved in the snow lotus plastome, including 4 rRNA, 25 tRNA, and 80 protein-coding genes. The snow lotus plastome included a 20-kb inversion and 3-kb translocation in the LSC region (Fig. S1). Such inversions have been observed in most Asteraceae (Zhang et al. 2019). No additional specific inversions were observed among the studied species. The IRb/SSC and IRa/LSC boundary shifts between the species commonly included the *ycf*1 and *rps*19 pseudogenes, respectively. Overall, our results demonstrated that the conservation pattern of plastomes is similar to those of other *Saussurea* species (Zhang et al. 2019; Yun and Kim 2022).

Many studies have reported the use of chloroplast SSR markers with high polymorphisms in *Saussurea* (Shen et al. 2020; Yun and Kim 2022). In previous studies, the genetic variability in snow lotus species has been investigated using genetic markers of the nuclear genome (Yuan et al. 2009; Wei et al. 2017; Nyamgerel et al. 2023), but they revealed lower genetic diversity. In this study, we found 49 SSRs (Fig. 3A), among these, 38 chloroplast SSRs ≥ 10 bp that can be used in population genetic studies (Table S8). These markers can aid in a better understanding of the genetic diversity and distribution patterns of snow lotus populations.

Previous phylogenetic studies of snow lotus species have mainly used nrDNA (ITS) and plastid (*mat*K, *rbc*L, *trn*K, *trn*H-*psb*A) barcoding markers (Chen and Wang 2018; Chen et al. 2019; Baasanmunkh et al. 2020). However, based on the plastome analysis of *Saussurea*, these plastid markers failed to resolve relationships across the genus (Zhang et al. 2019). Our study also revealed a relatively low nucleotide diversity in these four regions (Fig. 4). We identified two relatively variable regions (*ndh*J-*ndh*K and *ndh*D-*psa*C). Further barcoding studies of these two intergenic regions, combined with nuclear ITS markers, are needed to identify snow lotus species.

### Molecule evolution of the snow lotus group

Global cooling since the middle of the Miocene has affected *Saussurea* species in a wide variety of habitats, including cold and dry alpine meadows, steppe deserts, and screes (Xu et al. 2019; Zhang et al. 2021a). For example, the snow lotus species includes one of the most diverse groups within *Saussurea* that grow at high altitudes (Chen et al. 2019; Baasanmunkh et al. 2020). Understanding how different species adapt to extreme environments is an extension of the main goal of evolutionary biology (Zhang et al. 2021b). In this study, we estimated the selection pressure (Ka/Ks) of protein-coding genes in the snow lotus species, which has long been considered an indicator of adaptive evolution in green plants (Kimura 1979). Genes with a Ka/Ks > 1 ratio were considered under positive selection, and genes with a Ka/Ks ratio < 1 were considered under purifying selection (Yang and Bielawski 2000). Most protein-coding genes in *Saussurea* are under purifying selection (Zhang et al. 2019; Yun and Kim 2022). Among the snow lotus species, three genes were found to be under positive selection (Fig. 5). These genes may play important roles in the adaptation of the snow lotus to high altitudes. Among these, *ycf*2 is open reading frame in most higher plants that encodes proteins necessary for cell survival (Drescher et al. 2000). The *rpo*A gene encodes the α-subunit of RNA polymerase type I (plastid-encoded polymerase (PEP)) (Little and Hallick 1988). The *rpo* genes are relatively fast-evolving sequences that have been used as markers in phylogenetic studies (Krawczyk and Sawicki 2013). In addition, a photosynthesis-related gene, *ndh*B, was discovered in *S. baicalensis* and *S. dorogostaiskii*, which is associated with photosystem I to form a super-complex that mediates cyclic electron transport (Munekage et al. 2004).

Interestingly, the selective pressure on protein-coding genes in *S. dorogostaiskii* is similar to that in *S. baicalensis*, compared to other snow lotus species (Fig. 5). This result corresponds with the morphological features of *S. dorogostaiskii*. It is distinguished from the others by its elongated racemiform synflorescence and uppermost leaves, which are located on branches up to 1 m high (Table 2 and Fig. 1). *S. baicalensis* also exhibits racemiform synflorescences (Shi and Raab-Straube 2011). Other snow lotus species exhibit corymbiform synflorescences, which may be derived from racemiform synflorescences (Raab-Straube 2017). Furthermore, non-synonymous mutations of protein-coding genes were demonstrated in three types of patterns among the snow lotus species (Table S6): the first group was represented in *S. dorogostaiskii* and *S. baicalensis*, the second group was represented in *S. bogedaensis* and *S. involucrata*, and the third group included *S. orgaadayi* from China and Mongolia (Altai and Khangai Mountains). Within the second group, *S. involucrata* samples from China and Kyrgyzstan had various nucleotide substitutes, and *S. bogedaensis* (Mongolia) and *S. involucrata* (China) were relatively similar than *S. involucrata* (Kyrgyzstan). Further studies of the adaptation of the snow lotus species are required. In addition, *S. involucrata* (China) and *S. bogedaensis* (Mongolia) are most recently separated from *S. involucrata* (Kyrgyzstan) on the plastome analysis. They may share a common adaptation because of the similar conditions of environment as *S. involucrata* (Kyrgyzstan).

## Conclusion

The present study ascertained the nomenclature, phylogeny, and plastome evolution of the snow lotus species in China, Kyrgyzstan, Mongolia, and Russia. In particular, *S. involucrata* was excluded from the Mongolian flora, and *S. krasnoborovii* was synonymous with *S. dorogostaiskii*. The geographic distribution of each snow lotus was separated into the Altai, Tienshian, and Sayan Mountains. Snow lotus has a conserved plastome structure and gene content similar to those of most *Saussurea*. However, they have few genetic divergence regions and SSR markers that are suitable for identifying population genetic structure and gene flow patterns. In addition, *S. dorogostaiskii* is closely related to *S. baicalensis*, compared with other snow lotus species, and they have similar molecular adaptation patterns. This may have been caused by nonrandom recombination associated with climate change, making it an interesting topic for future evolutionary investigation.

### Supplementary Information

Below is the link to the electronic supplementary material.

Supplementary file1 (DOCX 1254 KB)

## Data Availability

The datasets generated and/or analyzed in this study are available in GenBank, National Center for Biotechnology Information (http://www.ncbi.nlm.nih.gov/genbank/) under the accession numbers of plastome: OR426625-OR426629 and ITS: OR673962, ON394565, ON399085, ON399084, ON399088, OQ826667, and OQ826670.
