# Supplementary material for: Comparative plastome analysis and taxonomic classification of snow lotus species (Saussurea, Asteraceae) in Central Asia and Southern Siberia
Source: Funct Integr Genomics. 2024 Feb 23;24(2):42. doi: 10.1007/s10142-024-01309-y (PMC10891264; doi:10.1007/s10142-024-01309-y)
Supplement: Supplementary file 1 — Supplementary file1 (DOCX 1254 KB) [file 10142_2024_1309_MOESM1_ESM.docx]

# **Supplementary Tables**

**Supplementary Table 1** Origins of plant materials used in this study.

| **Taxon** | **Origin** | **North latitude (*)** | **East longitude (*)** | **Altitude (m)** | **Specimen voucher** | **ITS accession no.** |
| --- | --- | --- | --- | --- | --- | --- |
| *Saussurea bogedaensis* | Mongolia, Bayankhongor province, Bogd soum, Ikh Bogd Mt. | 44.9397 | 100.379 | 3300 | KGZ38.1 | OR673962 |
| *S. orgaadayi* | Mongolia, Arkhangai province, Khangai soum, Ulzii mountain | 47.7777 | 99.2772 | 2408 | MGL004/KHA1 | ON394565 |
| *S. dorogostaiskii* | Mongolia, Khuvsgul province, Ulaan-Uul soum, Tag mountain | 50.5434 | 99.2349 | 2611 | UBU20180342 | ON399085 |
| *S. dorogostaiskii* | Mongolia, Khuvsgul province, Renchinlhumbe soum, Urtrag mountain | 50.4666 | 99.855 | 2486 | MNG001 | ON399084 |
| *S. dorogostaiskii* | Russian Federation, Republic of Tyva, Kaa-Khemsky district, Academiy Obruchev Ridge, Tumat-Taiga Mountains | 51.9764 | 95.4786 | 2241 | RUS002 | ON399088 |
| *S. involucrata* | Kyrgyzstan | 42.3686 | 79.0375 | 3605 | KYZ40-1-1 | OQ826667 |
| *S. baicalensis* | Mongolia, Khuvsgul province, Ulaan-Uul soum, Toomiin davaa | 50.4297 | 99.2218 | 2066 | KHU22-12-01 | OQ826670 |

**Supplementary Table 2** Raw reads and genome assembly information for the five sequenced samples.

|  |  | ***S. baicalensis*** | ***S. bogedaensis*** | ***S. dorogostaiskii*** | ***S. involucrata*** | ***S. orgaadayi*** |
| --- | --- | --- | --- | --- | --- | --- |
| Raw data | Total read bases | 9,362,978,480 | 8,484,023,788 | 8,753,600,766 | 9,694,050,812 | 8,516,396,376 |
|  | Total reads | 62,006,480 | 56,185,588 | 57,970,866 | 64,199,012 | 56,399,976 |
|  | Q20 (%) | 94.74 | 95.78 | 95.24 | 95.32 | 95.39 |
|  | Q30 (%) | 88.9 | 90.71 | 89.37 | 89.86 | 90 |
| Filtered | Trimmed reads (bp) | 6,766,006,624 | 6,581,198,668 | 6,572,813,096 | 7,287,337,585 | 6,422,691,088 |
|  | Trimmed bases (bp) | 45,133,842 | 43,842,670 | 43,832,592 | 48,567,706 | 42,803,480 |
|  | Q20 (%) | 98.95 | 99.06 | 98.97 | 99.01 | 99.02 |
|  | Q30 (%) | 96.04 | 96.41 | 95.85 | 96.24 | 96.25 |
| Assembly | Contig length | 152,624 | 152,513 | 152,624 | 152,512 | 152,594 |
|  | Number of contig | 1 | 1 | 1 | 1 | 1 |
|  | Mapped reads | 762,636 | 732,101 | 754,319 | 1,760,588 | 764,849 |
|  | Coverage (%) | 100 | 100 | 100 | 100 | 100 |
|  | Depth (X) | 688.98 | 661.37 | 682.27 | 1612.43 | 694,74 |

**Supplementary Table 3** Gene contents of the Snow Lotus species.

| **Category of genes** | **Group of genes** | **Name of genes** |
| --- | --- | --- |
| rRNA genes | | *rrn*16**^c^***, rrn*23**^c^***, rrn*4.5**^c^**, *rrn*5**^c^** |
| tRNA genes | | *trn*A-UGC**^a,c^**, *trn*C-GCA, *trn*D-GUC, *trn*E-UUC, *trn*F-GAA, *trn*G-GCC, *trn*G-UCC**^a^**, *trn*K-UUU**^a^**, *trn*H-GUG, *trn*I-CAU, *trn*I-GAU**^a^**, *trn*L-CAA**^c^**, *trn*L-UAG, *trn*L-UAA**^a^**, *trn*M-CAU**^c^**, *trn*M-CAU, *trn*M-CAU, *trn*N-GUU**^c^**, *trn*P-UGG, *trn*Q-UUG, *trn*R-ACG**^c^**, *trn*R-UCU, *trn*S-GCU, *trn*S-GGA, *trn*S-UGA, *trn*T-CGU, *trn*T-UGU, *trn*V-GAC^c^, *trn*V-UAC, *trn*W-CCA, *trn*Y-GUA |
| Self-replication | Small subunit of ribosome | *rps*2, *rps*3, *rps*4, *rps*7**^c^**, *rps*8, *rps*11, *rps*12 **^a,c,d^**, *rps*14, *rps*15, *rps*16 **^a^**, *rps*18, *rps*19 |
|  | Large subunit of ribosome | *rpl*16**^a^**, *rpl*14, *rpl*2**^a,c^***, rpl*20, *rpl*22, *rpl*23**^c^**, *rpl*32, *rpl*33, *rpl*36 |
|  | DNA dependent RNA polymerase | *rpo*A*, rpo*B*, rpo*C1*, rpo*C2**^a^** |
| Photosynthesis | Subunits of NADH-dehydrogenase | *ndh*A**^a^**, *ndh*B**^a^**^,^**^c^**, *ndh*C, *ndh*D, *ndh*E, *ndh*F, *ndh*G, *ndh*H, *ndh*I, *ndh*J, *ndh*K |
|  | Subunits of photosystem I | *psa*A*, psa*B*, psa*C*, psa*I*, psa*J |
|  | Subunits of photosystem II | *psb*A, *psb*B, *psb*C, *psb*D, *psb*E, *psb*F, *psb*H, *psb*I, *psb*J, *psb*K, *psb*L, *psb*M, *psb*N, *psb*T, *psb*Z*, ycf*3**^b^**, *ycf*4 |
|  | Subunits of cytochrome b/f complex | *pet*A, *pet*B**^a^**, *pet*D**^a^**, *pet*G, *pet*L, *pet*N |
|  | Subunits of ATP synthase | *atp*A**^a^**, *atp*B, *atp*E, *atp*F**^a^**, *atp*H, *atp*I |
|  | Large subunit of rubisco | *rbc*L |
| others | Maturase | *mat*K |
|  | Protease | *clp*P**^b^** |
|  | Envelope membrane protein | *cem*A |
|  | Subunit of Acetyl-CoA-carboxylase | *acc*D |
|  | c-type cytochrome synthesis gene | *ccs*A |
|  | Translational initiation factor | *inf*A |
| Genes of unknown functions | | *ycf*1*, ycf*2**^c^***, ycf*15 |
| **^a^**Gene with one intron, **^b^**Gene with two intron, **^c^**Gene with copies, **^d^**Trans-splicing gene | | |

**Supplementary Table 4** Intron-containing genes in Snow Lotus plastomes

| No. | **Gene** | **Region** | **Exon I** | **Intron I** | **Exon II** | **Intron II** | **Exon III** |
| --- | --- | --- | --- | --- | --- | --- | --- |
| 1 | *trn*K-UUU | LSC | 37 | 2462 | 35 |  |  |
| 2 | *rps*16 | LSC | 40 | 879 | 227 |  |  |
| 3 | *rpo*C1 | LSC | 432 | 728 | 1605 |  |  |
| 4 | *atp*F | LSC | 145 | 708 | 411 |  |  |
| 5 | *trn*G-UCC | LSC | 23 | 711 | 48 |  |  |
| 6 | *ycf*3 | LSC | 124 | 699 | 230 | 743 | 147 |
| 7 | *trn*L-UAA | LSC | 35 | 422 | 50 |  |  |
| 8 | *trn*V-UAC | LSC | 38 | 576 | 35 |  |  |
| 9 | *clp*P | LSC | 71 | 809 | 292 | 628 | 228 |
| 10 | *pet*B | LSC | 6 | 771 | 642 |  |  |
| 11 | *pet*D | LSC | 8 | 699 | 475 |  |  |
| 12 | *rpl*16 | LSC | 9 | 1106 | 399 |  |  |
| 13 | *rpl*2 | LSC | 391 | 665 | 434 |  |  |
| 14 | *ndh*B | IR | 777 | 670 | 756 |  |  |
| 15 | *rps*12 | (LSC) IR | (114) |  | 232 | 535 | 26 |
| 16 | *trn*I-GAU | IR | 38 | 940 | 35 |  |  |
| 17 | *trn*A-UGC | IR | 38 | 821 | 35 |  |  |
| 18 | *ndh*A | SSC | 551 | 1069 | 541 |  |  |

**Supplementary Table 5** Synonymous substitution (Ks) among the Snow lotus species and *S. baicalensis*.

|  |  | ***S. baicalensis* CHN** | ***S. baicalensis* MGL** | ***S. dorogostaiskii* MGL** | ***S. orgaadayi* CHN** | ***S. orgaadayi* MGL** | ***S. involucrata* KGZ** | ***S. bogedaensis* MGL** | ***S. involucrata* CHN** |
| --- | --- | --- | --- | --- | --- | --- | --- | --- | --- |
| Photosynthesis | *psa*A | 0.002 | 0.002 | 0.002 |  |  |  |  |  |
|  | *psa*B |  |  |  | 0.002 | 0.002 | 0.002 | 0.002 | 0.002 |
|  | *psb*B |  |  |  | 1.687 | 0.002 | 0.002 | 0.002 | 0.002 |
|  | *psb*C | 0.003 | 0.006 | 0.006 | 0.003 | 0.003 | 0.003 | 0.003 | 0.006 |
|  | *atp*A |  | 0.006 | 0.006 | 0.006 | 0.003 | 0.003 | 0.003 | 0.003 |
|  | *ndh*B | 2.088 | 2.088 | 2.088 |  |  |  |  |  |
|  | *ndh*D |  |  |  | 0.003 | 0.003 | 0.003 | 0.003 | 0.003 |
|  | *ndh*F |  |  |  | 2.598 |  |  |  |  |
|  | *ndh*I | 0.008 | 0.008 | 0.008 |  |  |  |  |  |
|  | *rbc*L | 0.010 | 0.010 | 0.010 | 0.007 | 0.007 | 0.007 | 0.007 | 0.010 |
| Self-replication | *rps*19 |  |  |  |  |  |  |  | 0.016 |
|  | *rps*2 | 0.007 |  |  |  |  |  |  |  |
|  | *rpo*A |  |  |  |  |  | 0.004 | 0.004 |  |
|  | *rpo*B | 0.003 | 0.003 | 0.003 | 0.003 | 0.006 | 0.003 | 0.003 | 0.005 |
|  | *rpo*C1 | 0.013 | 0.013 | 0.013 | 0.019 | 0.016 | 0.016 | 0.016 | 0.016 |
|  | *rpo*C2 | 4.975 | 4.975 | 4.975 | 0.002 | 0.002 | 0.003 | 0.003 | 0.002 |
|  | *mat*K |  | 0.002 | 0.002 | 0.003 |  |  |  |  |
|  | *acc*D | 0.012 | 0.012 | 0.012 | 0.005 |  |  |  |  |
|  | *ccs*A | 0.010 | 0.010 | 0.010 | 0.010 | 0.010 | 0.010 | 0.010 | 0.010 |
|  | *ycf*1 | 0.005 | 0.005 | 0.005 | 0.002 | 0.002 | 0.002 | 0.002 | 0.003 |
|  | *ycf*2 | 0.001 | 0.001 | 0.001 | 0.001 | 0.001 | 0.001 | 0.001 | 0.001 |
|  | *ycf*4 | 0.009 | 0.000 | 0.000 | 0.009 | 0.009 | 0.009 | 0.009 | 0.009 |

**Supplementary Table 6** Non-synonymous substitution (Ka) among the Snow lotus species and *S. baicalensis*.

|  |  | ***S. baicalensis* CHN** | | ***S. baicalensis* MGL** | | | ***S. dorogostaiskii* MGL** | | ***S. orgaadayi* CHN** | | ***S. orgaadayi* MGL** | | ***S. involucrata* KGZ** | | ***S. bogedaensis* MGL** | ***S. involucrata* CHN** |
| --- | --- | --- | --- | --- | --- | --- | --- | --- | --- | --- | --- | --- | --- | --- | --- | --- |
| Photosynthesis | *psa*A | | 0.001 | | 0.001 | 0.001 | | 0.001 | | 0.001 | | 0.001 | | 0.001 | | 0.001 |
|  | *psa*B | |  | |  |  | | 0.001 | | 0.001 | | 0.001 | | 0.001 | | 0.001 |
|  | *psa*C | | 0.006 | | 0.006 | 0.006 | | 0.011 | | 0.011 | | 0.011 | | 0.011 | | 0.011 |
|  | *psa*J | | 0.011 | | 0.011 | 0.011 | |  | |  | |  | |  | |  |
|  | *psb*A | | 0.001 | | 0.001 | 0.001 | | 0.001 | | 0.003 | | 0.003 | | 0.001 | | 0.001 |
|  | *psb*B | |  | |  |  | | 2.005 | | 0.001 | | 0.001 | | 0.001 | | 0.001 |
|  | *psb*C | |  | |  |  | |  | |  | |  | |  | |  |
|  | *psb*H | | 0.006 | | 0.006 | 0.006 | | 0.006 | | 0.006 | | 0.006 | | 0.006 | | 0.006 |
|  | *psb*J | | 0.011 | | 0.011 | 0.011 | |  | |  | |  | |  | |  |
|  | *psb*L | |  | |  |  | | 0.013 | | 0.013 | | 0.013 | | 0.013 | | 0.013 |
|  | *psb*T | |  | |  |  | | 0.014 | | 0.014 | | 0.014 | | 0.014 | | 0.014 |
|  | *atp*A | | 0.001 | | 0.001 | 0.001 | |  | |  | |  | | 0.001 | | 0.001 |
|  | *atp*B | | 0.002 | | 0.002 | 0.002 | | 0.001 | | 0.001 | | 0.001 | | 0.001 | | 0.001 |
|  | *atp*F | |  | |  |  | |  | | 0.002 | |  | |  | |  |
|  | *ndh*A | | 0.003 | | 0.001 | 0.001 | |  | |  | |  | |  | |  |
|  | *ndh*B | | 3.821 | | 3.821 | 3.821 | |  | |  | |  | |  | |  |
|  | *ndh*D | |  | |  |  | | 0.001 | | 0.001 | | 0.001 | | 0.002 | | 0.002 |
|  | *ndh*F | | 0.003 | | 0.005 | 0.005 | | 0.001 | | 0.001 | | 0.001 | |  | |  |
|  | *ndh*G | | 0.003 | |  |  | |  | |  | |  | |  | |  |
|  | *ndh*I | |  | |  |  | |  | |  | | 0.003 | |  | |  |
|  | *ndh*J | | 0.003 | | 0.003 | 0.003 | | 0.003 | | 0.006 | | 0.003 | | 0.003 | | 0.003 |
|  | *rbc*L | | 0.004 | | 0.004 | 0.004 | | 0.001 | | 0.001 | | 0.001 | | 0.001 | | 0.001 |
|  | *ycf*1 | | 0.006 | | 0.007 | 0.007 | | 0.005 | | 0.006 | | 0.006 | | 0.005 | | 0.005 |
|  | *ycf*2 | | 0.001 | | 0.001 | 0.001 | | 0.001 | | 0.001 | | 0.001 | | 0.001 | | 0.001 |
|  | *ycf*4 | | 0.002 | | 0.002 | 0.002 | |  | |  | |  | |  | |  |
| Self-replication | *rps*11 | | 0.003 | | 0.003 | 0.007 | | 0.007 | | 0.007 | | 0.007 | | 0.007 | | 0.007 |
|  | *rps*15 | | 0.011 | | 0.011 | 0.011 | |  | |  | |  | |  | |  |
|  | *rps*16 | | 0.011 | | 0.011 | 0.011 | |  | |  | |  | |  | |  |
|  | *rps*19 | |  | |  |  | |  | | 0.008 | | 0.008 | | 0.004 | | 0.004 |
|  | *rps*2 | | 0.002 | | 0.002 | 0.002 | |  | | 0.002 | | 0.002 | |  | |  |
|  | *rps*4 | | 0.002 | | 0.002 | 0.002 | | 0.002 | | 0.002 | | 0.002 | | 0.002 | | 0.002 |
|  | *rpl*16 | | 0.003 | |  |  | |  | |  | |  | |  | |  |
|  | *rpl*20 | | 0.004 | | 0.004 | 0.004 | |  | |  | |  | |  | |  |
|  | *rpl*22 | |  | |  |  | | 0.003 | | 0.003 | | 0.003 | | 0.003 | | 0.003 |
|  | *rpl*33 | | 0.007 | | 0.007 | 0.007 | |  | |  | |  | |  | |  |
|  | *rpl*36 | |  | |  |  | | 0.013 | |  | |  | |  | |  |
|  | *rpo*A | | 0.005 | | 0.007 | 0.007 | | 0.006 | | 0.006 | | 0.006 | | 0.007 | | 0.007 |
|  | *rpo*B | |  | |  |  | | 0.000 | |  | |  | |  | |  |
|  | *rpo*C1 | | 0.002 | | 0.002 | 0.002 | | 0.001 | | 0.001 | | 0.001 | | 0.001 | | 0.001 |
|  | *rpo*C2 | | 1.994 | | 1.994 | 1.994 | | 0.001 | | 0.001 | | 0.001 | | 0.002 | | 0.001 |
|  | *mat*K | | 0.003 | | 0.005 | 0.005 | | 0.002 | | 0.001 | | 0.001 | | 0.001 | | 0.001 |
|  | *cem*A | |  | |  |  | |  | |  | |  | | 0.002 | | 0.002 |
|  | *acc*D | | 0.006 | | 0.006 | 0.006 | | 0.004 | | 0.004 | | 0.004 | | 0.005 | | 0.004 |
|  | *ccs*A | | 0.001 | |  |  | |  | | 0.001 | |  | |  | |  |

**Supplementary Table 7** Detailed information on taxa, NCBI GenBank accession numbers.

| **Taxa** | **Subgenus** | **Section** | **GenBank accession number** | |
| --- | --- | --- | --- | --- |
|  |  |  | **Chloroplast** | **ITS** |
| *S. alata* | *Theodrea* | *Theodrea* |  | EF420929 |
| *S. alpina* | *Saussurea* | *Saussurea* |  | AF319091 |
| *S. amara* | *Theodrea* | *Theodrea* |  | EF420930 |
| *S. bogedaensis* | *Amphilaena* | *Amphilaena* |  | MF680702 |
| *S. bogedaensis* | *Amphilaena* | *Amphilaena* |  | MH209829 |
| *S. bogedaensis* | *Amphilaena* | *Amphilaena* | OR426627 | OR673962 |
| *S. baicalensis* | *Saussurea* | *Pycnocephala* | OR426628 | OQ826672 |
| *S. baicalensis* | *Saussurea* | *Pycnocephala* | MH926075 | AJ606207,  AJ606245 |
| *S. bracteata* | *Amphilaena* | *Amphilaena* | MH926080 | OR119909.1 |
| *S. dorogostaiskii* | *Saussurea* | *Pycnocephala* |  | ON399085 |
| *S. dorogostaiskii* | *Saussurea* | *Pycnocephala* |  | ON399084 |
| *S. dorogostaiskii* | *Saussurea* | *Pycnocephala* | OR426626 | ON399088 |
| *S. delavayi* | *Saussurea* | *Saussurea* |  | AB254648.1 |
| *S. globosa* | *Amphilaena* | *Amphilaena* |  | MF680676 |
| *S. glacialis* | *Eriocoryne* | *Eriocoryne* |  | AB118121 |
| *S. gnaphalodes* | *Eriocoryne* | *Eriocoryne* | MK953473 | MK225648.1 |
| *S. hypsipeta* | *Eriocoryne* | *Eriocoryne* |  | EF420919 |
| *S. involucrata* | *Amphilaena* | *Amphilaena* | OR426625 | OQ826672 |
| *S. involucrata* | *Amphilaena* | *Amphilaena* | MH926106 | MH003743 |
| *S. japonica* | *Theodrea* | *Theodrea* | MH926107 | AJ606172, AJ606212 |
| *S. krylovii* | *Saussurea* | *Pycnocephala* |  | AJ606205, AJ606243 |
| *S. laniceps* | *Eriocoryne* | *Eriocoryne* | MH926116 | OQ540356.1 |
| *S. leucophylla* | *Saussurea* | *Pycnocephala* | MH926118 | ON244070 |
| *S. medusa* | *Eriocoryne* | *Eriocoryne* | MN116789 | EF420918 |
| *S. obvallata* | *Amphilaena* | *Amphilaena* | MH926128 | MF680680 |
| *S. orgaadayi* | *Amphilaena* | *Amphilaena* | OR426629 | MT210907 |
| *S. orgaadayi* | *Amphilaena* | *Amphilaena* |  | ON394566 |
| *S. orgaadayi* | *Amphilaena* | *Amphilaena* |  | MH003774 |
| *S. schanginiana* | *Saussurea* | *Pycnocephala* |  | AJ606206, AJ606244 |
| *S. tridictyla* | *Eriocoryne* | *Eriocoryne* | MK953472 | OQ539999.1 |
| *Arctium lappa* |  |  | MH375874 | MH669232 |
| *Jurinea multiflora* |  |  |  | MH003704 |

**Supplementary Table 8** Detailed location of SSRs in Snow Lotus species and *S. baicalensis* plastomes.

|  | SSR | Location | ***S. baicalensis* MGL** | | ***S. dorogostaiskii* MGL** | | ***S. bogedaensis* MGL** | | ***S. involucrata* KGZ** | | ***S. orgaadayi* MGL** | |
| --- | --- | --- | --- | --- | --- | --- | --- | --- | --- | --- | --- | --- |
|  |  |  | start | end | start | end | start | end | start | end | start | end |
| 1 | (ATAA)3 | *trn*K-UUU intron | 1958 | 1969 | 1958 | 1969 | 1958 | 1969 | 1957 | 1968 | 1956 | 1967 |
| 2 | (A)10 | *trn*K-*rps*16 IGS | 4338 | 4350 | 4338 | 4350 | 4342 | 4351 | 4340 | 4349 | 4340 | 4350 |
| 3 | (C)13 | *rps*16 intron | 5309 | 5318 | 5309 | 5318 | 5317 | 5329 | 5313 | 5323 | 5313 | 5342 |
| 4 | (A)10 | *rpo*B | 13014 | 13023 | 13014 | 13023 | 13013 | 13022 | 13070 | 13079 | 13085 | 13094 |
| 5 | (A)10 | *rpo*C1 | 18057 | 18066 | 18057 | 18066 | 18056 | 18065 | 18113 | 18122 | 18128 | 18137 |
| 6 | (TA)5 | *rpo*C1 | 18280 | 18289 | 18280 | 18289 | 18279 | 18288 | 18336 | 18345 | 18351 | 18360 |
| 7 | (AT)5 | *rpo*C2 | 19276 | 19285 | 19276 | 19285 | 19275 | 19284 | 19332 | 19341 | 19347 | 19356 |
| 8 | (T)11 | *rpo*C2-*rps*2 IGS | 22868 | 22877 | 22868 | 22877 | 22873 | 22883 | 22930 | 22939 | 22945 | 22956 |
| 9 | (T)10 | *atp*I-*atp*H IGS | 25668 | 25677 | 25668 | 25677 | 25674 | 25683 | 25730 | 25740 | 24823 | 24833 |
| 10 | (AT)6 | *atp*H-*atp*F IGS | 26226 | 26237 | 26226 | 26237 | 26231 | 26242 | 26288 | 26299 | 26306 | 26319 |
| 11 | (TAA)4 | *trn*G-UCC intron | 30700 | 30711 | 30700 | 30711 | 30708 | 30719 | 30766 | 30777 | 30782 | 30793 |
| 12 | (TTC)4 | *psb*C | 34170 | 34181 | 34170 | 34181 | 34182 | 34193 | 34239 | 34250 | 34255 | 34266 |
| 13 | (A)10 | *psb*C-*trn*S IGS | 34357 | 34368 | 34357 | 34368 | 34369 | 34378 | 34426 | 34435 | 34442 | 34451 |
| 14 | (A)10 | *psbC-trnS IGS* | 35292 | 35303 | 35292 | 35303 | 35302 | 35311 | 34441 | 34450 | 34457 | 34467 |
| 15 | (A)12 | *ycf*3-*trn*S IGS | 43652 | 43669 | 43652 | 43669 | 43659 | 43670 | 43716 | 43730 | 43736 | 43747 |
| 16 | (T)13 | *ndh*C-*trn*V IGS | 49784 | 49794 | 49784 | 49794 | 49749 | 49761 | 49798 | 49810 | 49825 | 49840 |
| 17 | (T)11 | *atp*B-*rbc*L IGS | 54139 | 54150 | 54139 | 54150 | 54094 | 54104 | 54138 | 54150 | 54168 | 54181 |
| 18 | (AAT)4 | *acc*D-*psa*I IGS | 58457 | 58468 | 58457 | 58468 | 58372 | 58383 | 58418 | 58429 | 58449 | 58460 |
| 19 | (T)10 | *psa*I-*ycf*4 IGS | 58809 | 58818 | 58809 | 58818 | 58724 | 58733 | 58770 | 58780 | 58801 | 58810 |
| 20 | (T)12 | *psb*E-*pet*L IGS | 64540 | 64552 | 64540 | 64552 | 64471 | 64482 | 64522 | 64534 | 64557 | 64570 |
| 21 | (TA)7 | *rpl*33-*rps*18 IGS | 67290 | 67301 | 67290 | 67301 | 67221 | 67234 | 67273 | 67286 | 67309 | 67322 |
| 22 | (TATT)3 | *rpl*33-*rps*18 IGS | 67346 | 67357 | 67346 | 67357 | 67279 | 67290 | 67331 | 67342 | 67367 | 67378 |
| 23 | (A)10 | *rpl*20-*rps*12 IGS | 68564 | 68573 | 68564 | 68573 | 68497 | 68506 | 68549 | 68558 | 68585 | 68594 |
| 24 | (ATA)4 | *clp*P1 intron 2 | 70632 | 70643 | 70632 | 70643 | 70566 | 70577 | 70619 | 70630 | 70654 | 70665 |
| 25 | (A)11 | *pet*D-*rpo*A IGS | 77268 | 77277 | 77268 | 77277 | 77204 | 77214 | 77257 | 77266 | 77292 | 77301 |
| 26 | (T)17 | *rpo*A | 77514 | 77530 | 77514 | 77530 | 77450 | 77466 | 77502 | 77511 | 77537 | 77547 |
| 27 | (T)14 | *rps*8-*rpl*14 IGS | 79946 | 79963 | 79946 | 79963 | 79882 | 79895 | 79934 | 79947 | 79969 | 79982 |
| 28 | (T)10 | *rpl*14-*rpl*16 IGS | 80536 | 80545 | 80536 | 80545 | 80469 | 80478 | 80521 | 80530 | 80557 | 80567 |
| 29 | (A)12 | *rrn*5-*trn*R IGS | 107050 | 107062 | 107050 | 107062 | 106970 | 106981 | 107017 | 107028 | 107053 | 107063 |
| 30 | (T)12 | *trn*R-*trn*N IGS | 107513 | 107525 | 107513 | 107525 | 107432 | 107443 | 107479 | 107489 | 107514 | 107525 |
| 31 | (TTTA)3 | *ndh*F-*rpl*32 IGS | 111719 | 111730 | 111719 | 111730 | 111642 | 111653 | 111688 | 111699 | 111724 | 111735 |
| 32 | (T)13 | *rpl*32-*trn*L IGS | 112828 | 112846 | 112828 | 112846 | 112750 | 112762 | 112797 | 112806 | 112832 | 112845 |
| 33 | (AGAA)3 | *ndh*A intron 1 | 119534 | 119545 | 119534 | 119545 | 119455 | 119466 | 119506 | 119517 | 119542 | 119553 |
| 34 | (AATC)3 | *ndh*A intron 1 | 119941 | 119952 | 119941 | 119952 | 119863 | 119874 | 119913 | 119924 | 119949 | 119960 |
| 35 | (GATT)3 | *ycf*1 | 124385 | 124396 | 124385 | 124396 | 124289 | 124300 | 124294 | 124305 | 124375 | 124386 |
| 36 | (CAAA)3 | *ycf*1 | 127071 | 127082 | 127071 | 127082 | 126975 | 126986 | 126980 | 126991 | 127061 | 127072 |
| 37 | (A)12 | *trn*N-*trn*R IGS | 128642 | 128654 | 128642 | 128654 | 128546 | 128557 | 128551 | 128561 | 128632 | 128643 |
| 38 | (T)12 | *trn*R-*rrn*5 IGS | 129105 | 129117 | 129105 | 129117 | 129008 | 129019 | 129012 | 129023 | 129094 | 129104 |

# **Supplementary Figures legend**

**
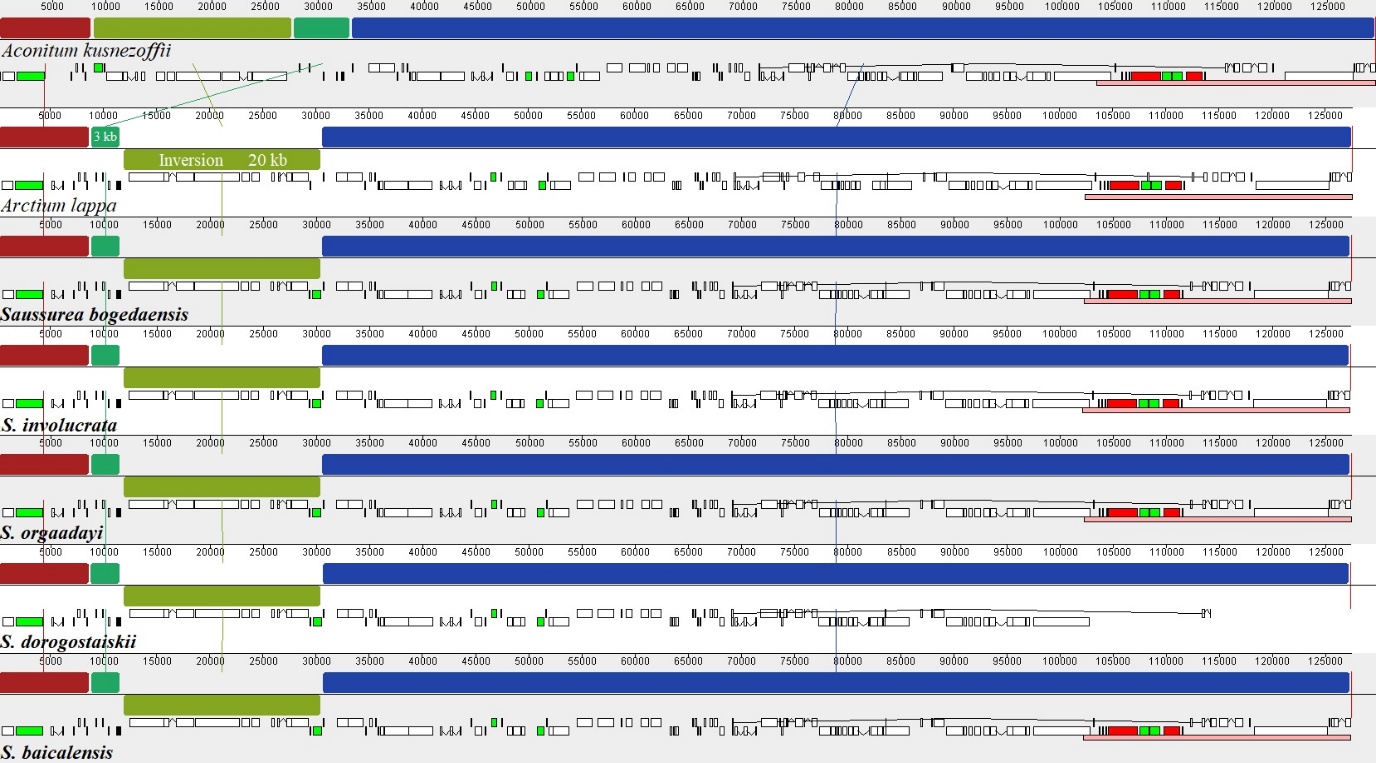
Supplementary Fig. 1** MAUVE alignment of the plastome of five newly sequenced *Saussurea* species with those of *Arctium lappa* (Asteraceae) and *Aconitium kusnezoffii* (Ranunculaceae). The blocks in the top row are in the same orientation, whereas those in the bottom row are in inverse orientation. Boxes in each genome map represent protein-coding genes (white), rRNAs (red), and tRNAs (green).

**
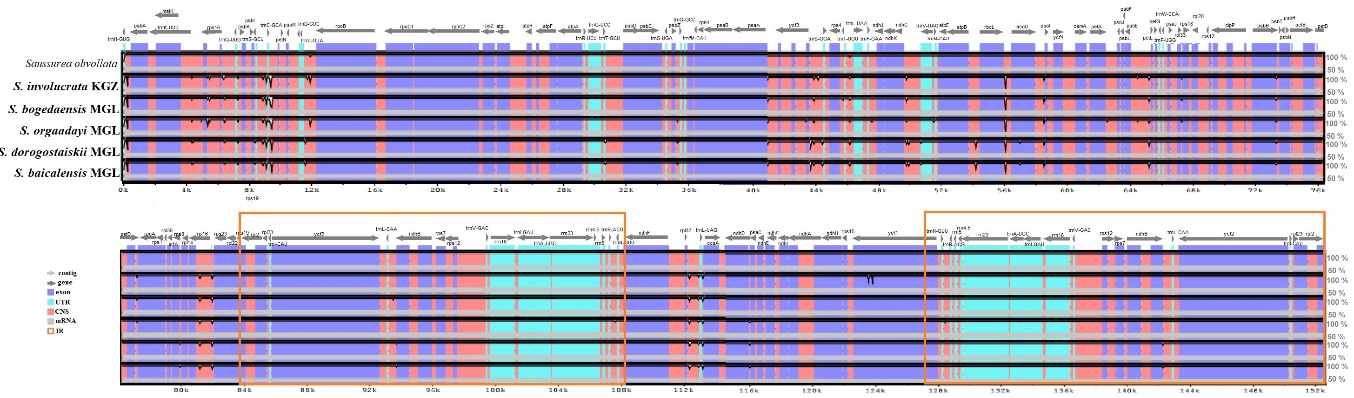
 Supplementary Fig. 2** Comparison of Snow Lotus species and *S. baicalensis* plastomes. Gray arrows above the alignment indicate the genes. The horizontal axis indicates coordinates within the plastome. Gray arrows above the alignment indicate the extension direction of the gene; purple indicates the exon, blue indicates the untranslated region, pink indicates the non-coding sequences, and gray indicates mRNA. The vertical scale represents the percent identity, ranging from 50 to 100%. The newly sequenced samples are in bold.

**
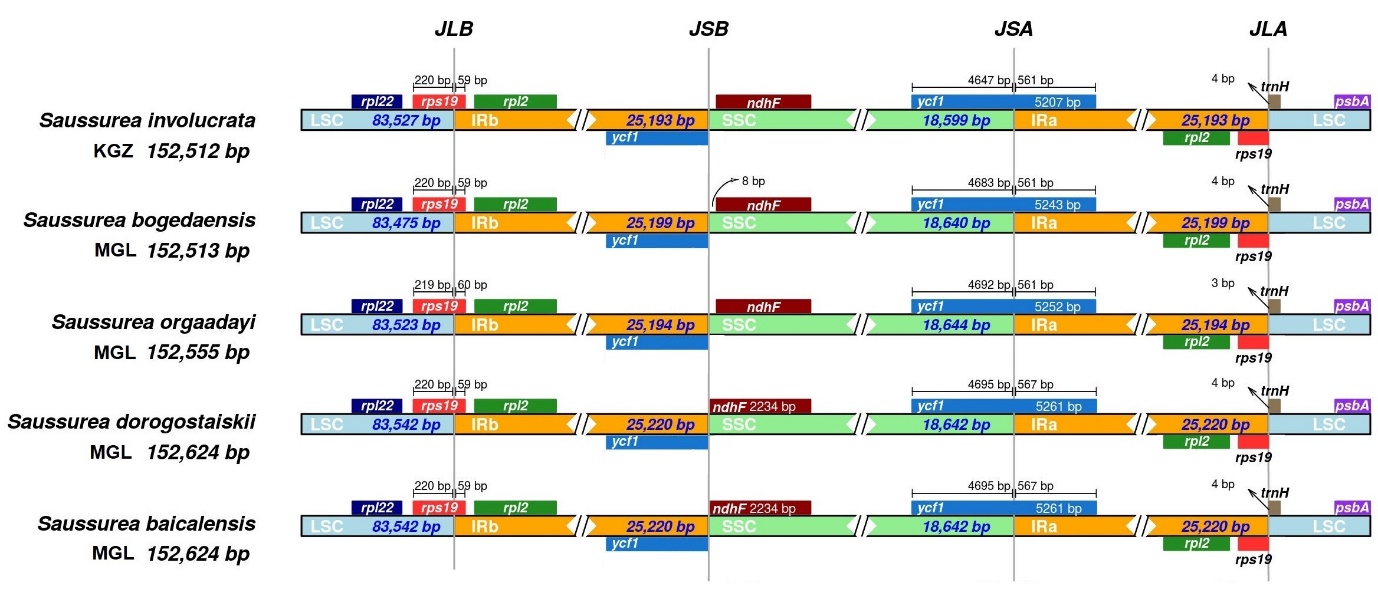
Supplementary Fig. 3** Comparison of LSC, IR, and SSC junction positions in Snow Lotus species and *S. baicalensis* plastomes. LSC, large single-copy region; SSC, small single-copy region; IR, inverted repeat region.

**
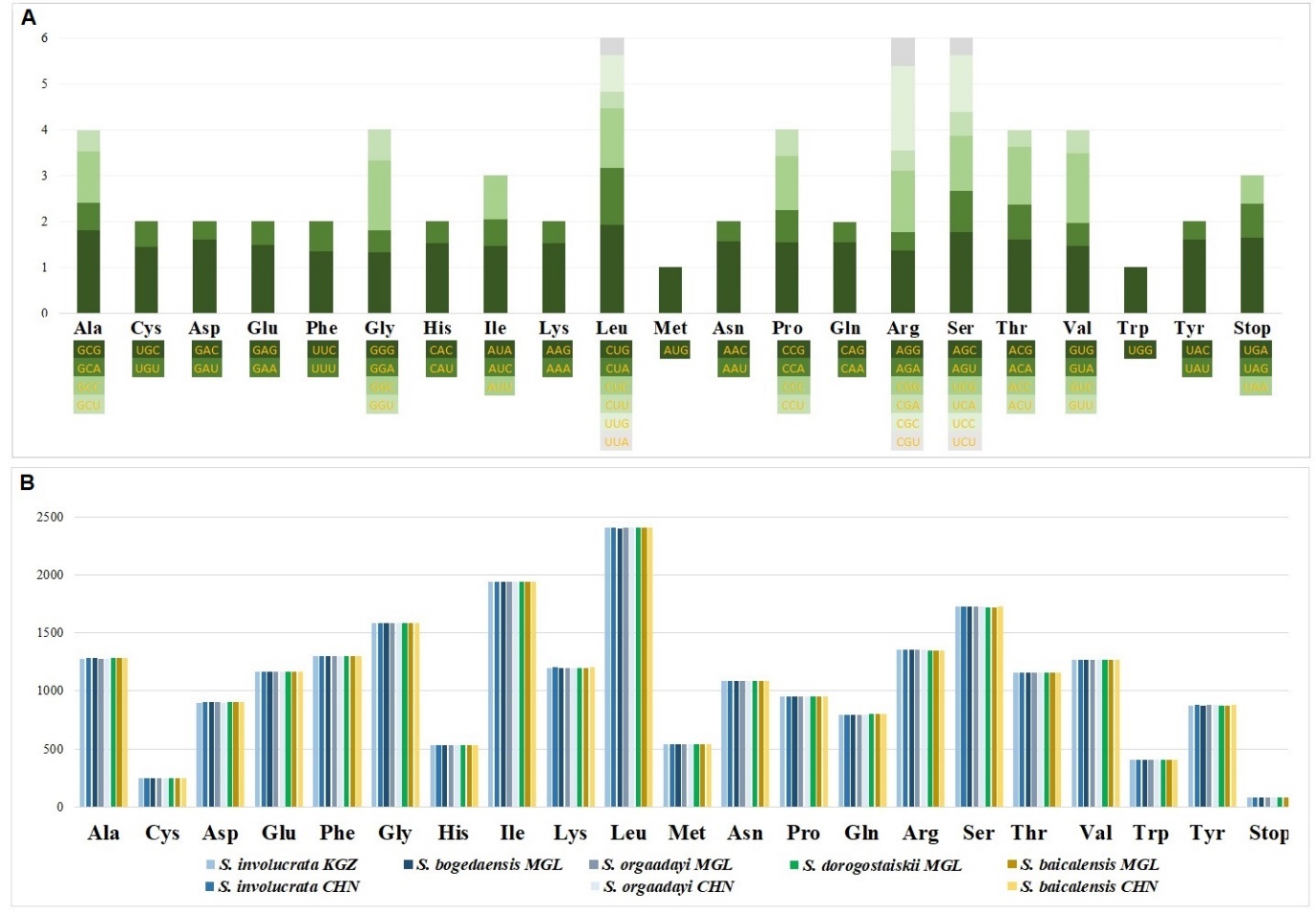
Supplementary Fig. 4** Codon usage and anticodon recognition patterns of the Snow lotus plastome. (**A**) Codon usage of 20 amino acids in the protein-coding genes. Colors in the column graph reflect codons in the same color as shown below the figure. RSCU: relative synonymous codon usage. (**B**) Amino acid frequencies in the protein-coding sequences.

**
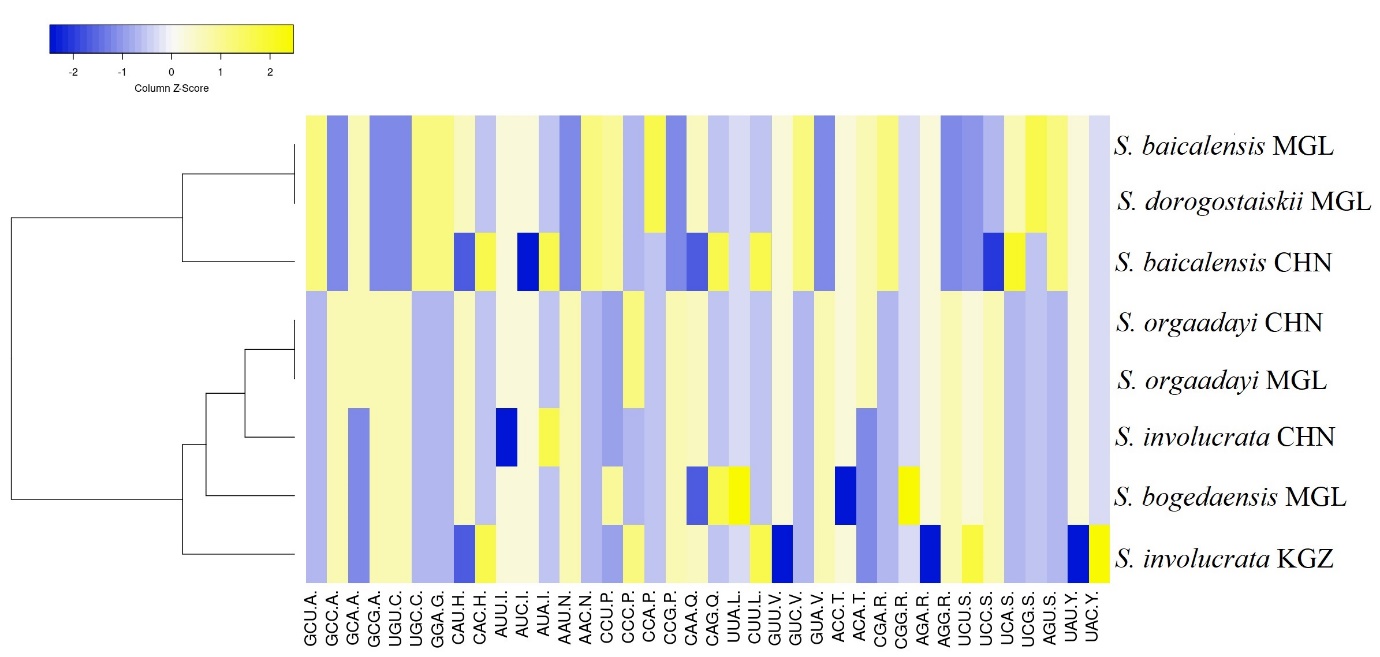
Supplementary Fig. 5** Heatmap of the Snow Lotus species and *S. baicalensis* plastomes. Yellow indicates a high relative synonymous codon usage (RSCU) value and blue indicates a low RSCU value. Hierarchical clustering (average linkage method) was performed based on the codon patterns (x-axis)
